# A new synthetic access to 2-*N*-(glycosyl)thiosemicarbazides from 3-*N*-(glycosyl)oxadiazolinethiones and the regioselectivity of the glycosylation of their oxadiazolinethione precursors

**DOI:** 10.3762/bjoc.9.16

**Published:** 2013-01-21

**Authors:** El Sayed H El Ashry, El Sayed H El Tamany, Mohy El Din Abdel Fattah, Mohamed R E Aly, Ahmed T A Boraei, Axel Duerkop

**Affiliations:** 1 HEJ Research Institute of Chemistry, International Center for Chemical and Biological Sciences, Karachi University, Karachi, Pakistan; 2 Chemistry Department, Faculty of Science, Alexandria University, Alexandria, Egypt; 3 Chemistry Department, Faculty of Science, Suez Canal University, Ismailia, Egypt; 4 Chemistry Department, Faculty of Applied Science, Port Said University, Port Said, Egypt; 5 Institute of Analytical Chemistry, Chemo and Biosensors, Universitätsstrasse 31, 93053 Regensburg, Germany

**Keywords:** glycosyloxadiazolinethiones, glycosylsulfanyloxadiazoles, glycosylthiosemicarbazides, thermal rearrangement, X-ray crystallography

## Abstract

Glycosylations of 5-(1*H*-indol-2-yl)-1,3,4-oxadiazoline-2(3*H*)-thione delivered various degrees of *S*- and/or *N*-glycosides depending on the reaction conditions. *S*-Glycosides were obtained regiospecifically by grinding oxadiazolinethiones with acylated α-D-glycosyl halides in basic alumina, whereas 3-*N*-(glycosyl)oxadiazolinethiones were selectively obtained by reaction with HgCl_2_ followed by heating the resultant chloromercuric salt with α-D-glycosyl halides in toluene under reflux. On using Et_3_N or K_2_CO_3_ as a base, mixtures of *S*- (major degree) and *N*-glycosides (minor degree) were obtained. Pure 3-*N*-(glycosyl)oxadiazolinethiones can also be selectively obtained from glycosylsulfanyloxadiazoles by the thermal *S*→*N* migration of the glycosyl moiety, which is proposed to occur by a tight-ion-pair mechanism. Thermal *S*→*N* migration of the glycosyl moiety can be used for purification of mixtures of *S*- or *N*-glycosides to obtain the pure *N*-glycosides. The aminolysis of the respective *S*- or *N*-glycosides with ammonia in aqueous methanol served as further confirmation of their structures. While in *S*-glycosides the glycosyl moiety was cleaved off again, 3-*N*-(glycosyl)oxadiazolinethiones showed a ring opening of the oxadiazoline ring (without affecting the glycosyl moiety) to give *N*-(glycosyl)thiosemicarbazides. Herewith, a new synthetic access to one of the four classes of glycosylthiosemicarbazides was found. The ultimate confirmation of new structures was achieved by X-ray crystallography. Finally, action of ammonia on benzylated 3-*N*-(galactosyl)oxadiazolinethione unexpectedly yielded 3-*N*-(galactosyl)triazolinethione. This represents a new path to the conversion of glycosyloxadiazolinethiones to new glycosyltriazolinethione nucleosides, which was until now unknown.

## Introduction

Modified nucleosides are versatile motifs for studying the relationship between the structure and functions of nucleic acids and problems of metabolism, besides their main potential in curing viral infections and cancer diseases [1]. The 1,3,4-oxadiazolines and 1,2,4-triazolines are potential inhibitors of physiologically relevant isoforms of the zinc enzyme carbonic anhydrase (CA, EC 4.2.2.1), i.e., cytosolic CA I and CA II, the tumor-associated transmembrane isoenzyme CA IX with inhibition constants in the low micromolar range [2]. Coupling of aglycones with relevant glycosyl donors is the common approach involved in the synthesis of most nucleosides. Another strategy for the synthesis of nucleoside analogues is the use of glycosylamines and related *N*-bonded glycosides [3–7], such as glycosylisothiocyanates, which afford glycosylthiosemicarbazides [8] or glycosyl 3-thioureidothiourea derivatives [9]. These 4-*N*-(glycosyl)thiosemicarbazides [8] were used for the synthesis of Schiff-like bases [10] and 4-*N*-glycosyl(thiosemicarbazido)phosphorothionates as precursors for the synthesis of the herbicidal and fungicidal agents thiazolidine-4-ones [11].

Thiosemicarbazide (TSC) and related amines were used to prepare modified amylase and amylopectin for biological studies [12]. Moreover, glycosylthiosemicarbazides are formed in vivo to modify cell-surface sialic acid. This is known as a metabolic cell-surface-engineering technique for cell-surface interactions and consequently shows the potential of these compounds for the development of anticancer agents [13–16]. Antituberculosis effects of glycosylthiosemicarbazides were also reviewed [17]. Glycosylamines are used also as enzyme inhibitors and vaccine precursors [18–21], and in glycopeptide synthesis [22–23] and in glycodendrimers and glycoclusters [24–25].

**Scheme 1:**
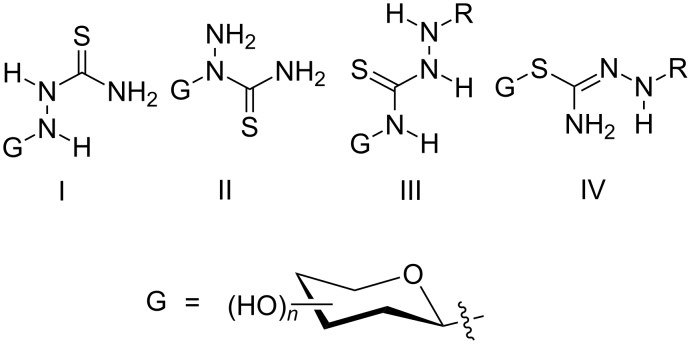
Structures of the different glycosylthiosemicarbazides.

There are four structural isomers of glycosyl-thiosemicarbazides according to the location of the glycosyl residue on the thiosemicarbazide; the 1-*N*-, 2-*N*-, *S*- and 4-*N*- (glycosyl)thiosemicarbazides (Scheme 1). To the best of our knowledge, examples of 1-*N*-(**I**)-type [26] and 4*-N*-(**III**)-type glycosylthiosemicarbazides [27–31] are known. While we had focused on simple alkylations and glycosylations of 1,3,4-oxadiazolethione [32–33] and on 2-*N*-(glycosyl)thiosemicarbazides **II** obtained from the aminolysis of 3-*N*-(glycosyl)oxadiazolinethione precursors, the growing interest in glycosylthiosemicarbazides stimulated the development of direct regioselective formations of the corresponding 3-*N*-(glycosyl)oxadiazolinethiones, which are shown in this work. Earlier literature showed that 3-*N*-(glycosyl)oxadiazolinethiones could be obtained from the chloromercuric salts of 1,3,4-oxadiazolethiones by heating at high temperatures in dry nitromethane under reflux using dry calcium sulfate [34] or toluene [35–37]. In continuation of our previous work [33] in which the synthesis of compounds **5** to **13** and **20** were published in a preliminary manner without the experimental details, this paper introduces more examples for the thermal rearrangement of *S*-glycosides to the corresponding *N*-glycosides, which deliver excellent yields without solvent or a catalyst in a short reaction time. Moreover, the thiosemicarbazide **II** precursors can also be selectively obtained from 3-*N*-(glycosyl)oxadiazolinethiones.

In addition to our previous work [33], we investigated the potential of the benzylated *N*-glycosides as precursors for the respective thiosemicarbazides. Surprisingly, the formation of the galactosyl-1,2,4-triazoline-3-thione from the galactosyl-1,3,4-oxadiazole-2(3*H*)-thione was discovered for the first time. This is a new method for the conversion of an 1,3,4-oxadiazole-2(3*H*)-thione ring into a 1,2,4-triazoline-3-thione, because formerly, only conversions of 1,3,4-oxadiazolethione (by reaction of hydrazine hydrate) to 4-amino-1,2,4-triazolinethiones were reported [38–40]. The products of the new reactions are verified by the X-ray single-crystal analysis of the galactosyl-1,2,4-triazoline-3-thione, and a representative glucopyranosylsulfanyl-1,3,4-oxadiazole and glucopyranosylthiosemicarbazide, each.

## Results and Discussion

**Scheme 2:**
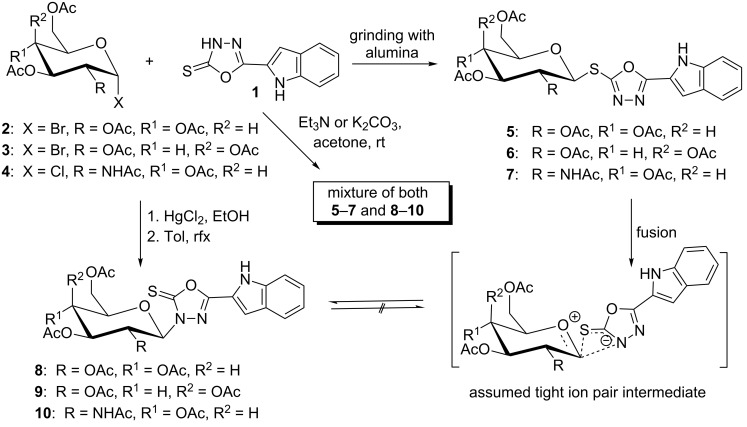
Glycosylations of oxadiazolinethione.

The regiospecificity of glycosylations of 1,3,4-oxadiazolinethiones was tested by reacting 5-(1*H*-indol-2-yl)-1,3,4-oxadiazoline-2(3*H*)-thione (**1**) with a set of α-D-glycosyl halides **2–4** under different conditions. Glycosylations in the presence of either Et_3_N or K_2_CO_3_ yielded a mixture of both the *S*- (**5**–**7**) and *N*-linked (**8**–**10**) glycosides in varying yields of 42–71% and 12–35%, respectively (Scheme 2). Generally, both bases could be considered more regioselective towards *S*-glycosides than towards the *N*-glycosyl analogues. Grinding the reactants with basic alumina afforded regiospecifically *S*-linked glycosides **5–7** in 52–63% yields. However, if glycosylations were carried out on the chloromercuric salt of **1** in toluene under reflux, 3-*N*-linked glycosides **8–10** were regiospecifically obtained in 48–60% yields.

Anomeric β-configurations of the *S*-linked **5–7** and *N*-linked **8–10** glycosides were deduced from the ^1^H NMR spectra, which revealed large *J*_1,2_ values of 10.3–10.6 and 9.2–9.5 Hz, respectively, for the anomeric protons. The chemical shifts of the anomeric protons of *S*-glycosides were at lower values (δ 5.45–5.56 ppm) than those of *N*-glycosides (δ 5.92–6.10 ppm). The differentiation between *S*- and *N*-glycosides was supported by the presence or absence of the signal of the carbon atom of the C=S moiety in the ^13^C NMR spectra. In other words, the ^13^C NMR spectra of the *N*-glycosides **8–10** revealed signals at δ_c_ 176.10–177.40 ppm. Anomeric carbons in both types were observed at δ 83.20–84.00 ppm.

Thermal rearrangement of the *S*-glycosides **5–7** under solvent-free and atmospheric conditions afforded the corresponding 3*-N-*glycosides **8–10**. The conversion was achieved in a few minutes with good to excellent yields (60–90%). Therefore, the thermal rearrangement from *S*- to *N*-glycosides may also serve as a rapid and economic (free of solvents) purification step for crude mixtures of *S*- and 3-*N*-glycosides obtained from glycosylations mediated by either Et_3_N or K_2_CO_3_. Additional experiments on crude mixtures of *S*- and 3-*N*-glycosides successfully afforded pure *N*-glycosides.

The mechanism of this rearrangement is presumably proceeding by an ionization–recombination pathway in which a thermally induced heterolysis of the thioglycosidic bond results in a tight ion pair generated upon ionization of this bond. An intramolecular reaction of the tight ion pair results in migration of the glycosyl moiety from sulfur to nitrogen, which proceeds with complete retention of configuration. As a result, a tight-ion-pair mechanism in which the migrating group retains chirality is suggested (Scheme 2).

**Scheme 3:**
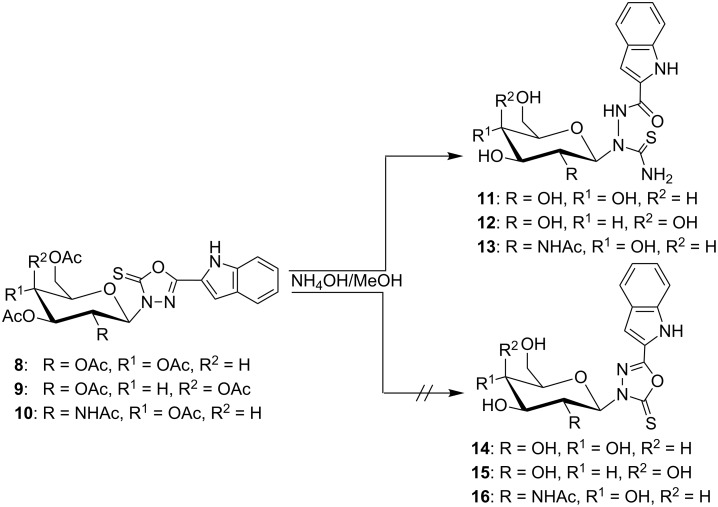
Synthesis of glycosylthiosemicarbazides by solvolysis.

Aminolysis of the *N*-glycosides **8–10** (Scheme 3) mediated with ammonia in aqueous methanolic solution led to de-*O*-acetylation of the glycan moieties along with aminolysis of the oxadiazole ring affording 2-*N*-(glycosyl)thiosemicarbazides **11–13** instead of the corresponding nucleosides **14–16**. The oxadiazole ring cleavage combined with de-*O*-acetylation of **8–10** is proven by three facts. First, the molecular weights derived from the mass spectra of the products **11–13** are higher by seventeen atomic mass units than would be those of the deacetylated products **14–16**. Second, the IR spectra of **11–13** show new amide absorption bands that do not appear in the IR spectra of their precursors. Finally, the ^13^C NMR spectra of **11–13** show signals for NC=O groups at δ_C_ 159.40–162.90 ppm in addition to the NC=S groups at δ_C_ 183.10–184.50 ppm. Deacetylation of **10** is only confined to the *O*-acetyl groups, but the *N-*acetyl group survived under these conditions. This was confirmed by an extra ^13^C NMR signal at δ_C_ 172.50 ppm for the NH*C*OCH_3_ group of **13**. The presence of ^1^H NMR signals at δ_H_ 6.46–6.55 ppm as doublets with coupling constants of *J*_1,2_ = 8.5–9.0 Hz indicated the stability of the pyranose ring and its β-anomeric configuration under these conditions.

**Scheme 4:**
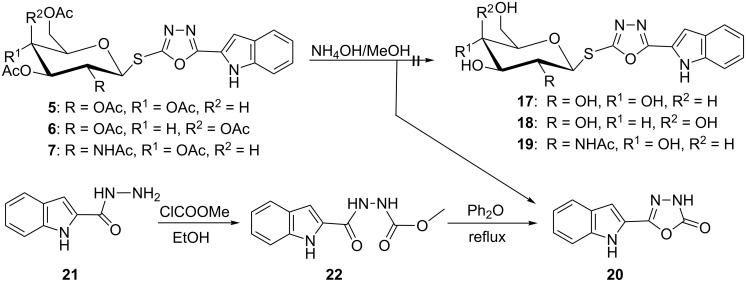
Solvolysis of glycosylsulfanyloxadiazoles.

Aminolysis of the *S*-glycosides **5–7** (Scheme 4) under the same conditions (ammonia in aqueous methanolic solution) generally led to splitting of the thioglycosyl moiety as a result of hydrolysis of the bond between the oxadiazole (C_2_) and the glycosidic sulfur atom. Hence, the indolyloxadiazolone **20** was formed [33] whereas the expected deacetylated glycosides **17–19** were not obtained. Structure elucidation of **20** yielded a melting point of 271–273 °C [33], while literature reports give a value of 102 °C [41] or 285 °C [42]. Therefore, compound **20** was prepared in another reaction sequence [33] to prove its structure and the correctness of the physical and structural data obtained. Hence, the indol-2-carbohydrazide (**21**) was reacted with methyl chloroformate followed by cyclization of the resulting ester **22**. As a result, **20** was obtained in high yield, and its structural analytical data were identical with those of the aminolysis product (Scheme 4).

**Scheme 5:**
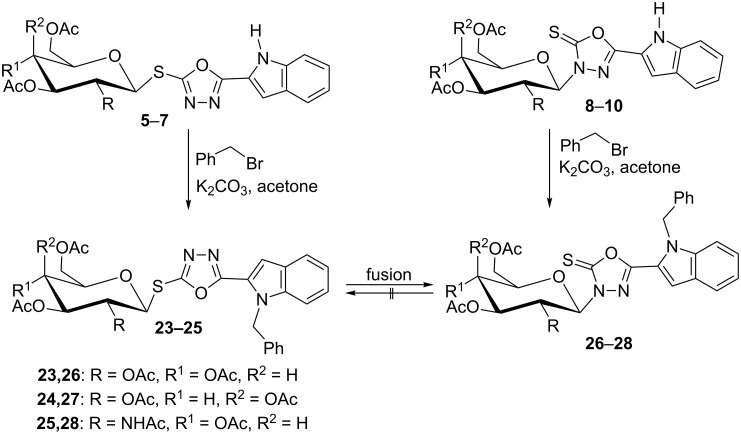
Benzylation of the *S*- and *N*-glycosides **5–10**.

For ultimate confirmation of the structures of *S*- and *N*-glycosides **5–10**, the benzylation of the indole was chosen, because these derivatives can serve to prove the glycosyl rearrangement and glycosyl TSC formation. Moreover, crystalline glycosides are obtained that are suitable for X-ray analysis. In the presence of K_2_CO_3_, the *S*- and *N*-glycosides **23–28** (Scheme 5) were obtained. Disappearance of the NH-signal of the indole in the ^1^H NMR spectra of all products and appearance of the benzyl methylene protons at δ_H_ 5.76–5.97 ppm and the methylene carbon at δ_C_ 48.4–48.8 ppm in the ^13^C NMR spectra support a successful benzylation. Additional NMR signals of the phenyl protons and carbon atoms of the phenyl ring were another strong evidence for the *N*-benzylation of the indole units. The different (yet large) values of the coupling constants (*J*_1,2_) of the anomeric protons of *S*-glycosides **23–25** (10–10.4 Hz) and of the *N*-glycosides **26–28** (9.5–9.6 Hz), respectively, indicate that all benzylated glycosides still have the β-configuration. Successful thermal *S*→*N* glycosyl migration (and the concomitant change of structural analytical data of the products) served as a final proof that *N*-glycosylated products were obtained from their former pure *S*-analogues.

**Scheme 6:**
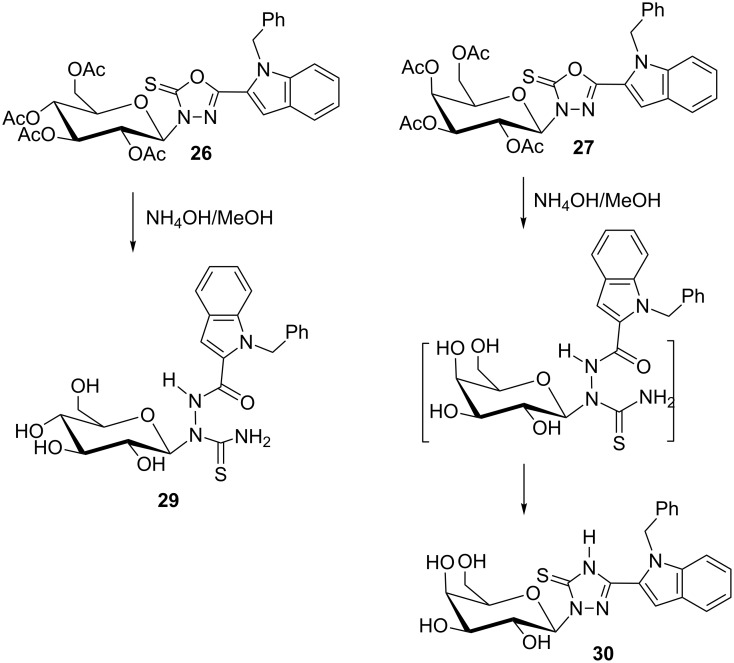
Aminolysis of benzylated indolyl-3-*N*-(glucosyl)- and (galactosyl)oxadiazolinethiones.

Aminolysis of the benzylated *N*-glycosides **26** and **27** was done with ammonia in aqueous methanolic solution to obtain additional new thiosemicarbazide derivatives. Although, the reaction conditions remained unchanged compared to the cleavage of **8–10**, derivatives **26** and **27** (Scheme 6) yielded different products under these conditions. Oxadiazole ring cleavage combined with de-*O*-acetylation converted **26** into the corresponding 2-*N*-(glycosyl)thiosemicarbazide **29** while the galactonucleoside **27** was converted into the galactosyltriazole **30**. We propose that it is formed by a cyclization with the elimination of water from a thiosemicarbazide as intermediate. The structure of **30** (and other key compounds) could be confirmed by X-ray crystallography. The large values of the coupling constants (*J*_1,2_) of the anomeric protons of the *N*-glycosides **29**, **30** indicate that *β*-configuration is still retained here.

## X-ray analysis

**Figure 1:**
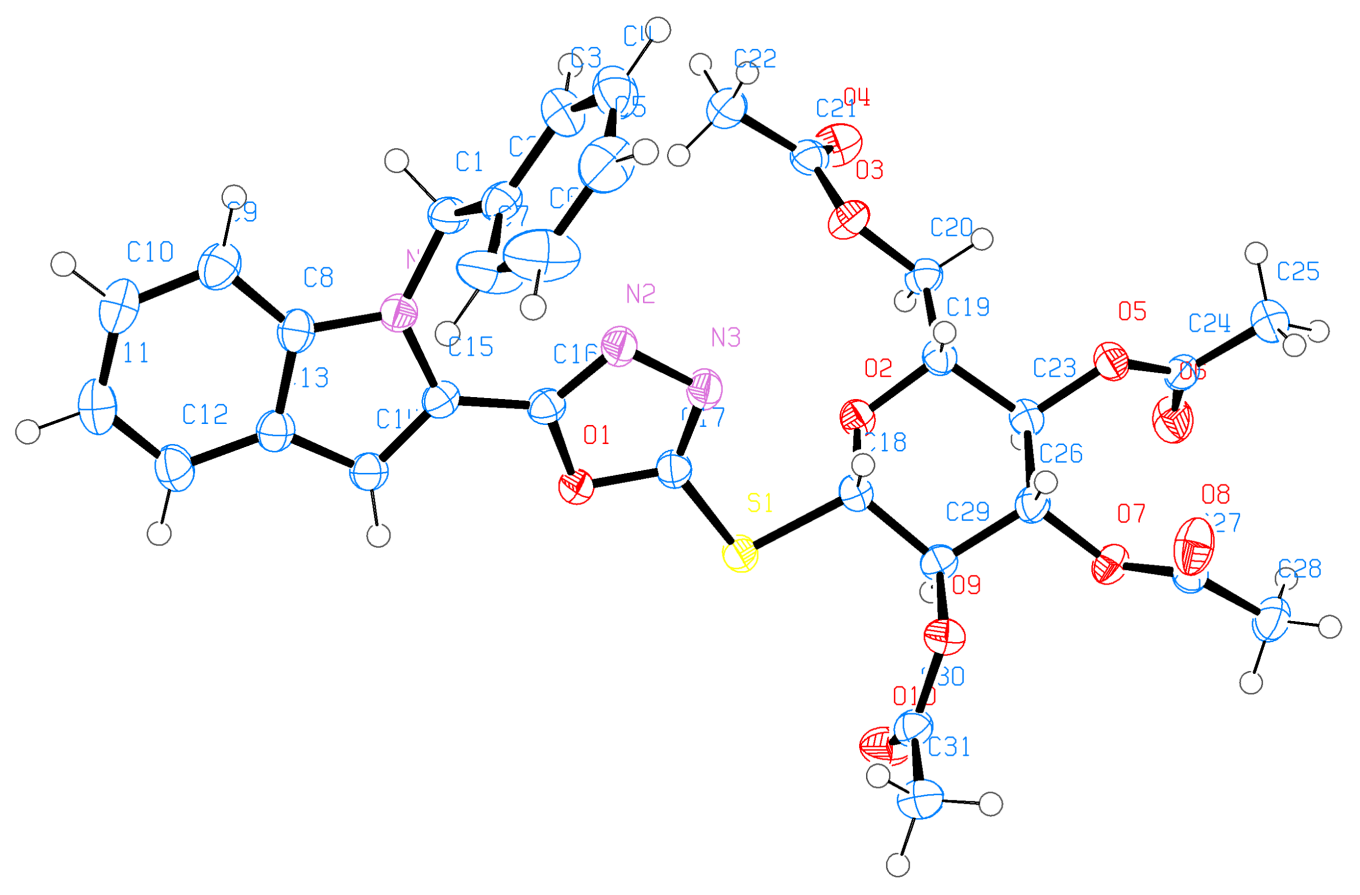
ORTEP representation of **23**.

**Figure 2:**
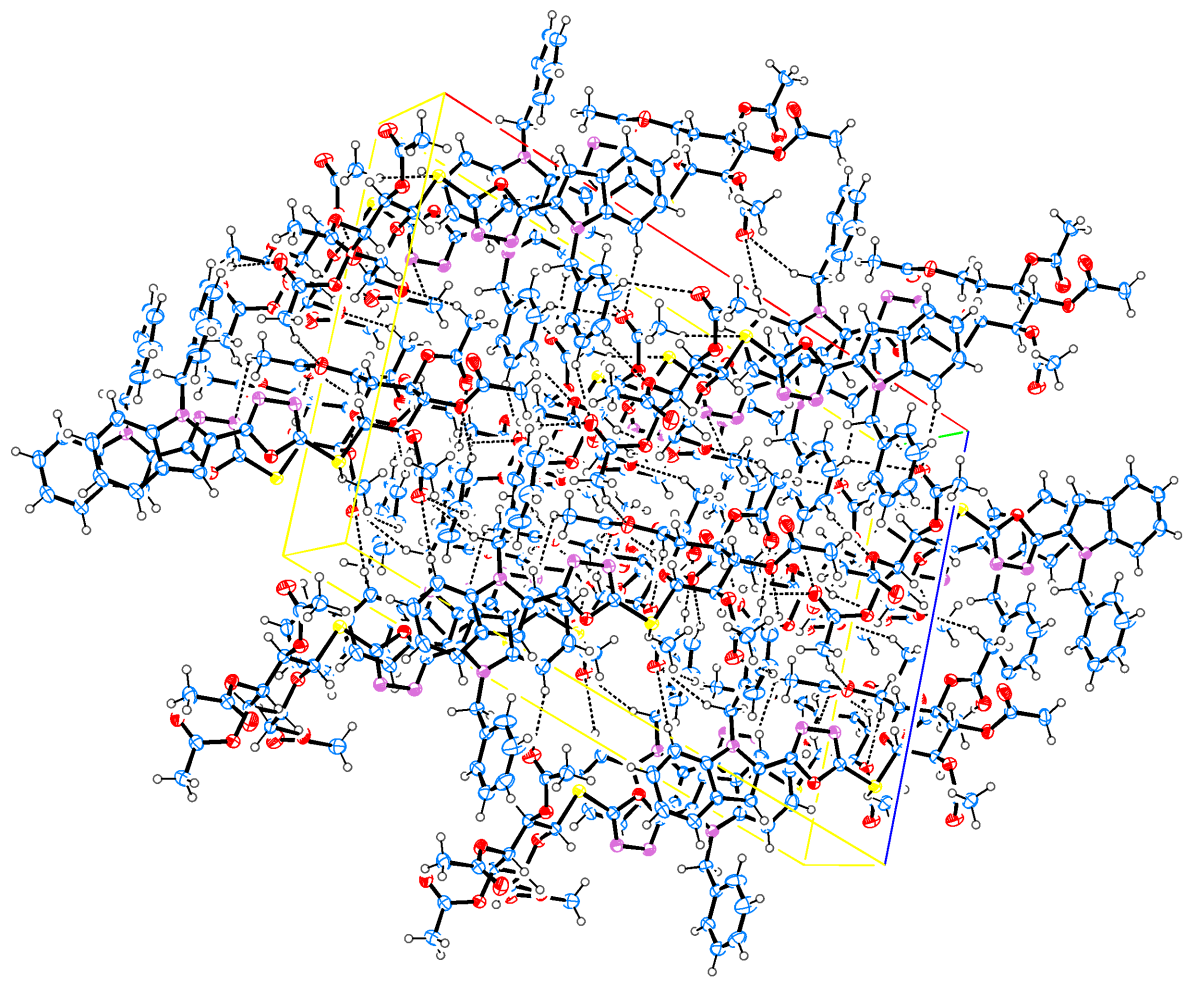
Packing diagram of **23**.

Single-crystal X-ray diffraction experiments yielded unambiguous confirmations of the structural assignments of the *S*-glycoside **23**, the 2-*N*-(glycosyl)thiosemicarbazide **29**, and the galactosyltriazole **30**. Single crystals were slowly grown in EtOH. **23** crystallized in the monoclinic space group C_2_ with the following unit cell parameters: *a* = 25.7078 Å, α = 90º, *b* = 7.1500 Å, β = 105.2576º, *c* = 17.8411 Å, γ = 90º and *V* = 3163.81 Å^3^. The crystallographic data of **23** are shown in Table 1. The whole molecule is nonplanar; the phenyl group is located perpendicular to the plane of the indole ring by making a torsion angle of C(8)N(1)C(1)C(2) = 95.9º while the oxadiazole ring is located in the plane of the indole ring by making torsion angles of N(1)C(15)C(16)O(1) = −171.48°, C(14)C(15)C(16)O(1) = 10.6º and N(1)C(15)C(16)N(2) = 9.2°, respectively. The crystallographic analysis revealed that the sugar molecule has the glucopyranose form and has ^4^*C*_1_ conformation. The anomeric β-configuration is derived from the bond lengths of O(2)–C(18) and O(2)–C(19), which are 1.416 and 1.442 Å, and all substituents have equatorial orientation. Moreover, the crystal data revealed that the S(1)–C(17) bond length is 1.743 Å suggesting a certain degree of conjugation with the oxadiazole ring, whereas the S(1)–C(18) bond length is 1.816 Å, which is typical for single bonds of this kind [43]. The crystal structure and molecular conformation is stabilized by three intramolecular C–H···N hydrogen bonds, three intramolecular C–H···O hydrogen bonds, and five intermolecular C–H···O hydrogen bonds in the crystal network (Figure 1, Figure 2 and Table 1).

**Table 1:** Crystal data, instrumental and refinement data for **23**.

Crystal data	

Empirical formula	C_31_H_31_N_3_O_10_S
Formula weight	637.66
Crystal size	0.2241 × 0.0456 × 0.0328 mm
Crystal description	Stick
Crystal color	Colorless
Crystal system	Monoclinic
Space group	*C*_2_
Unit-cell dimensions	*a* = 25.7078 Å; α = 90° *b* = 7.15002 Å; β = 105.2576° *c* = 17.8411 Å; γ = 90°
Volume	3163.81(8) Å^3^
*Z*	4
Calculated density	1.339 Mg/m^3^
Absorption coefficient	1.433 mm^−1^
F(000)	1336

Data collection	

Measurement device type	SuperNova, single source at offset, Atlas
Measurement method	w Scans
Temperature	123 K
Wavelength	1.54184 Å
Monochromator	Graphite
Theta range for data collection	3.56 to 73.15°
Index ranges	−31<=*h*<=31, −8<=*k*<=7, −22<=*l*<=22
Reflections collected/unique	11616/4707 [R(int) = 0.0199]
Reflections greater	I>2\s(I);4572
Absorption correction	Analytical
Max. and min. transmission	0.960 and 0.833

Refinement	

Refinement method	Full-matrix least-squares on F^2^
Hydrogen treatment; Data/restraints/parameters	4707/1/406
Goodness-of-fit on F^2^	1.040
Final R indices [I>2sigma(I)]	R1 = 0.0305, wR2 = 0.0817
R indices (all data)	R1 = 0.0314, wR2 = 0.0827
Absolute structure parameter	0.024(14)
Largest diff. peak and hole	0.286 and −0.282 *e*·Å^−3^
	

**Figure 3:**
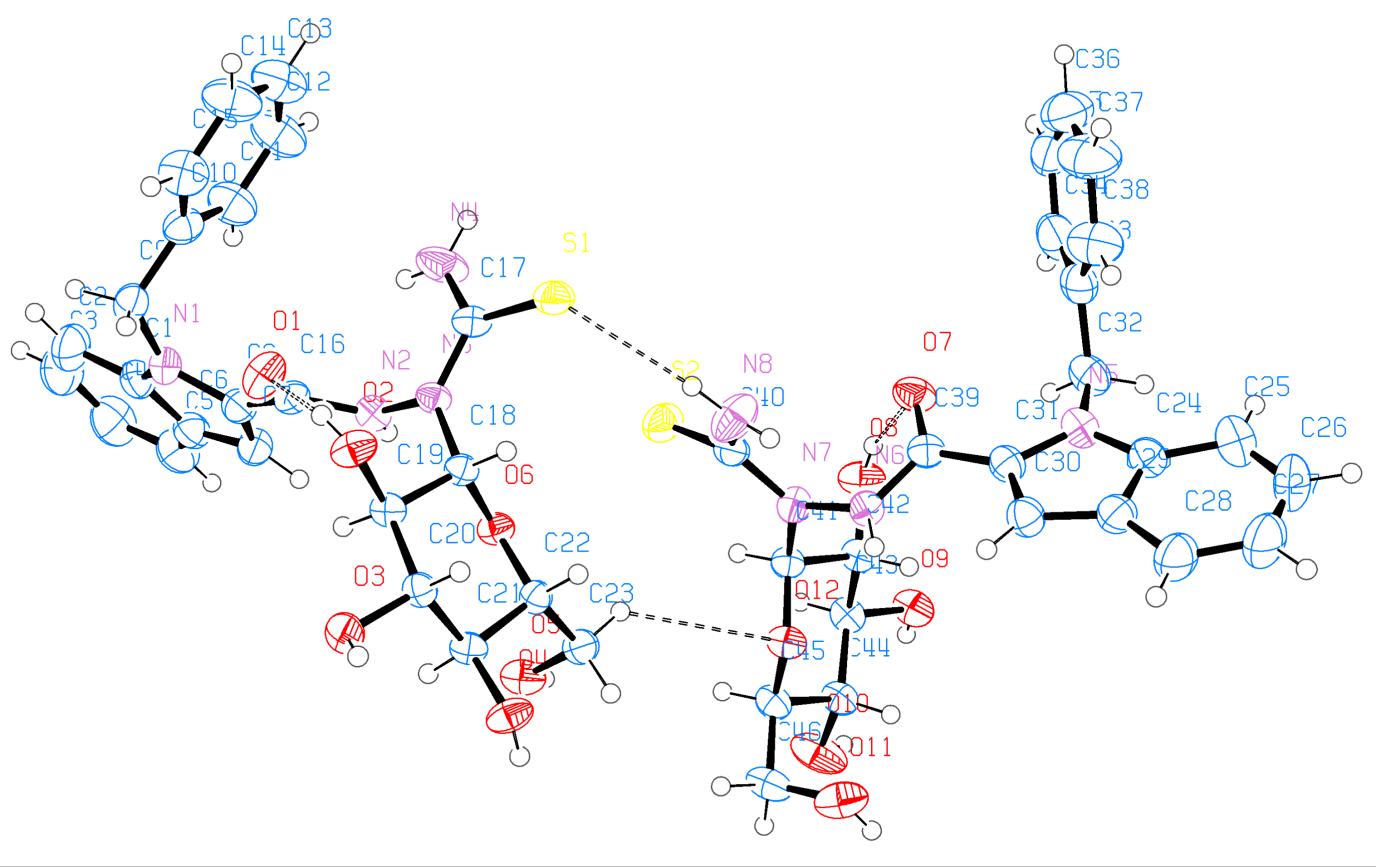
ORTEP representation of **29**.

**Figure 4:**
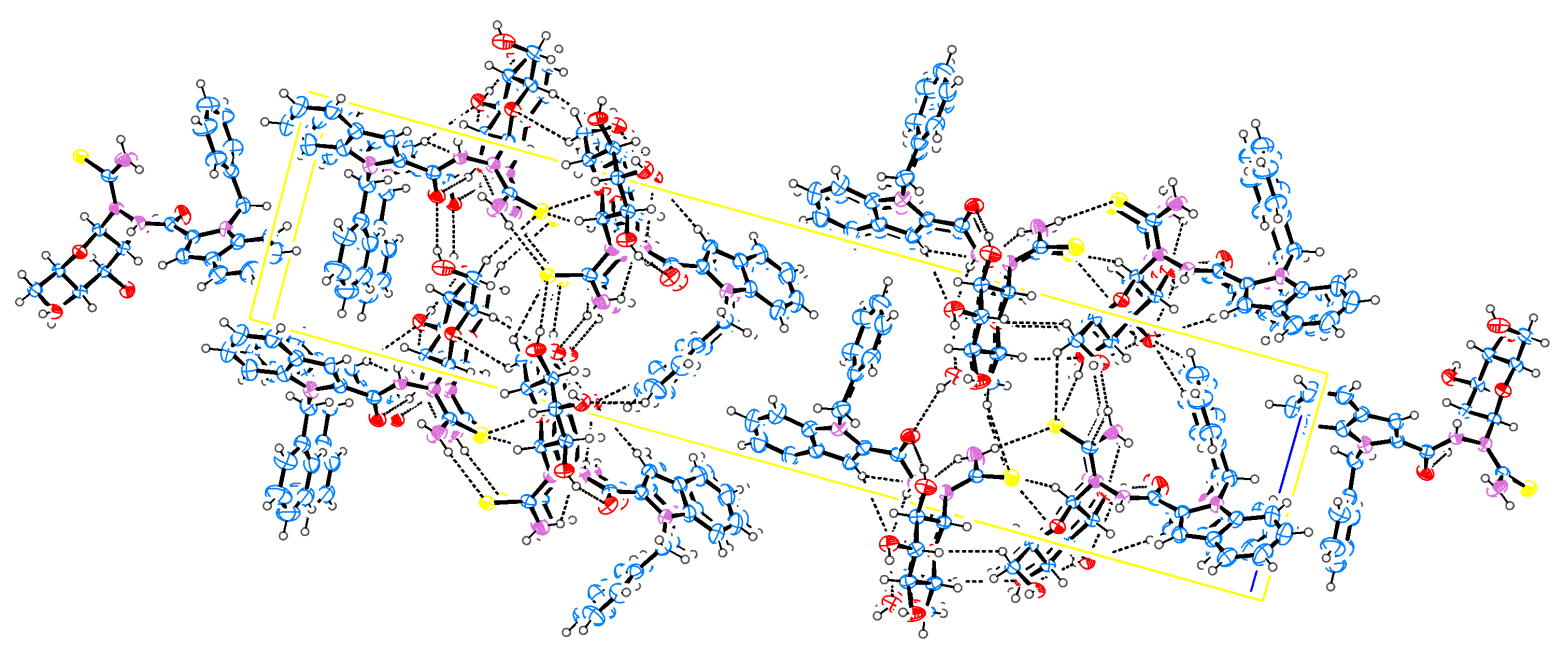
Packing diagram of **29**.

Compound **29** (Figure 3, Figure 4 and Table 2) crystallized as a cyclic dimer and contains two independent molecules in the unit cell. The dimer is stabilized by an intermolecular hydrogen bond N(8)–H(8M)···S(1). Moreover, S(1)–C(17) and S(2)–C(40) displayed bond lengths of 1.699 and 1.660 Å, respectively, reflecting the double-bond character of the thiocarbonyl unit of the TSC group. This also supports the cleavage of the oxadiazole ring in the reaction of **26** to **29**. The bond lengths O(6)–C(18) and O(6)–C(22) are 1.414 and 1.433 Å (similar to those found in **23**), respectively, which shows that the sugar moiety still has the glucopyranose form with ^4^C_1_ anomeric β-configuration. In addition, the crystal structure of **29** shows that the whole molecule is nonplanar. The phenyl group makes a dihedral angle of C(1)N(1)C(9)C(10) = −100.3°, which means that it is perpendicular to the indole ring.

**Table 2:** Crystal data, instrumental and refinement data for **29**.

Crystal data	

Empirical formula	C_23_H_26_N_4_O_6_S
Formula weight	486.55
Crystal size	0.3575 × 0.0513 × 0.0329 mm
Crystal description	Needle
Crystal color	Colorless
Crystal system	Monoclinic
Space group	*P*2_1_
Unit-cell dimensions	*a* = 6.25643 Å; α = 90° *b* = 41.019 Å; β= 96.092° *c* = 9.1898 Å; γ = 90°
Volume	2345.06 Å^3^
*Z*	4
Calculated density	1.378 Mg/m^3^
Absorption coefficient	1.632 mm^−1^
F(000)	1024

Data collection	

Measurement device type	SuperNova, single source at offset, Atlas
Measurement method	w Scans
Temperature	293 K
Wavelength	1.54184 Å
Monochromator	Graphite
Theta range for data collection	4.31 to 73.83°
Index ranges	−4<=*h*<=7, −50<=*k*<=50, −11<=*l*<=10
Reflections collected/unique	8866/7227 [R(int) = 0.0371]
Reflections greater	I>2\s(I) 6676
Absorption correction	Analytical
Max. and min. transmission	0.953 and 0.749

Refinement	

Refinement method	Full-matrix least-squares on F^2^
Hydrogen treatment; Data/restraints/parameters	7227/1/633
Goodness-of-fit on F^2^	1.009
Final R indices [I>2sigma(I)]	R1 = 0.0431, wR2 = 0.1113
R indices (all data)	R1 = 0.0463, wR2 = 0.1132
Absolute structure parameter	0.054(15)
Largest diff. peak and hole	0.798 and −0.275 *e*·Å^−3^

**Figure 5:**
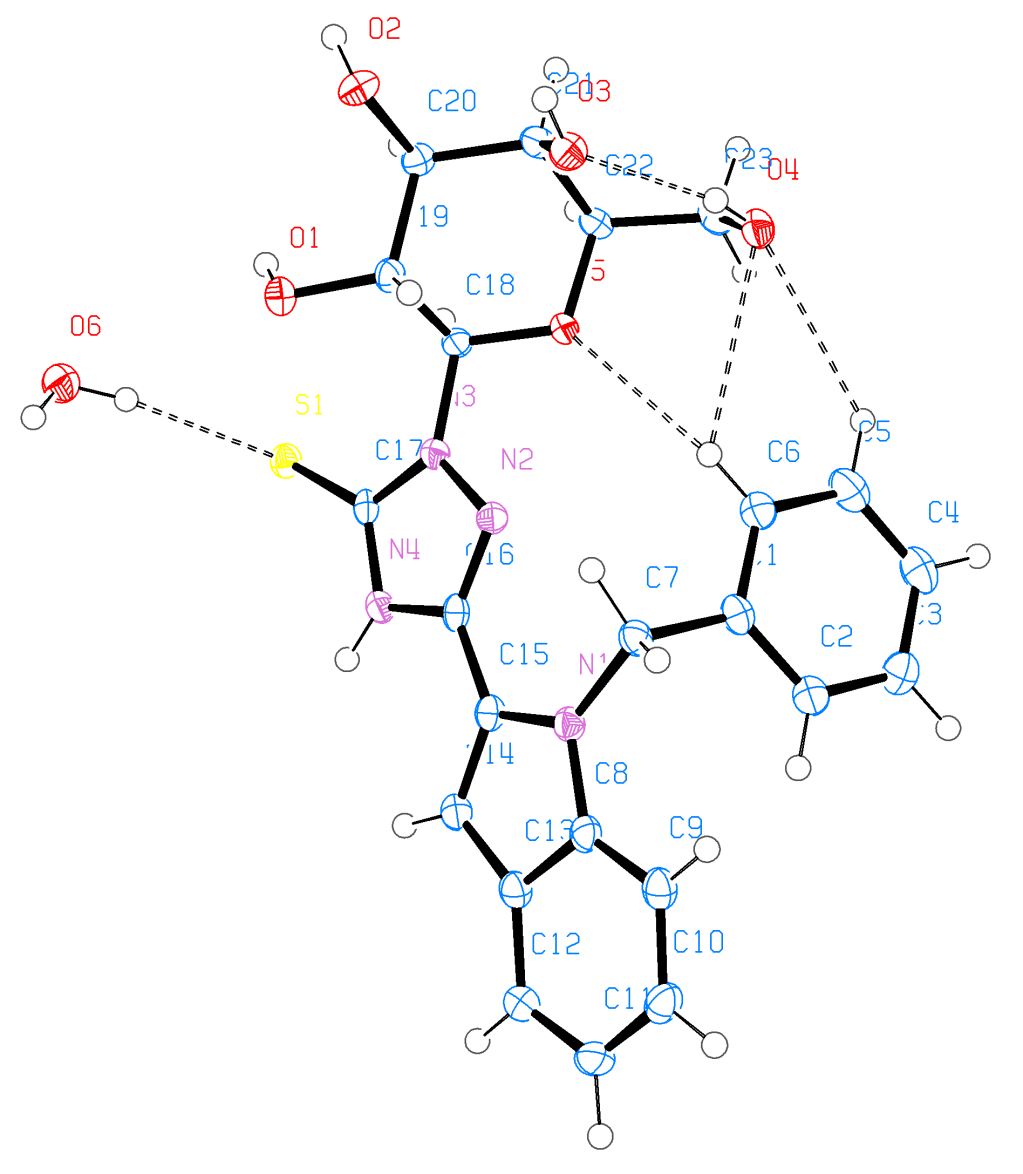
ORTEP representation of **30**.

**Figure 6:**
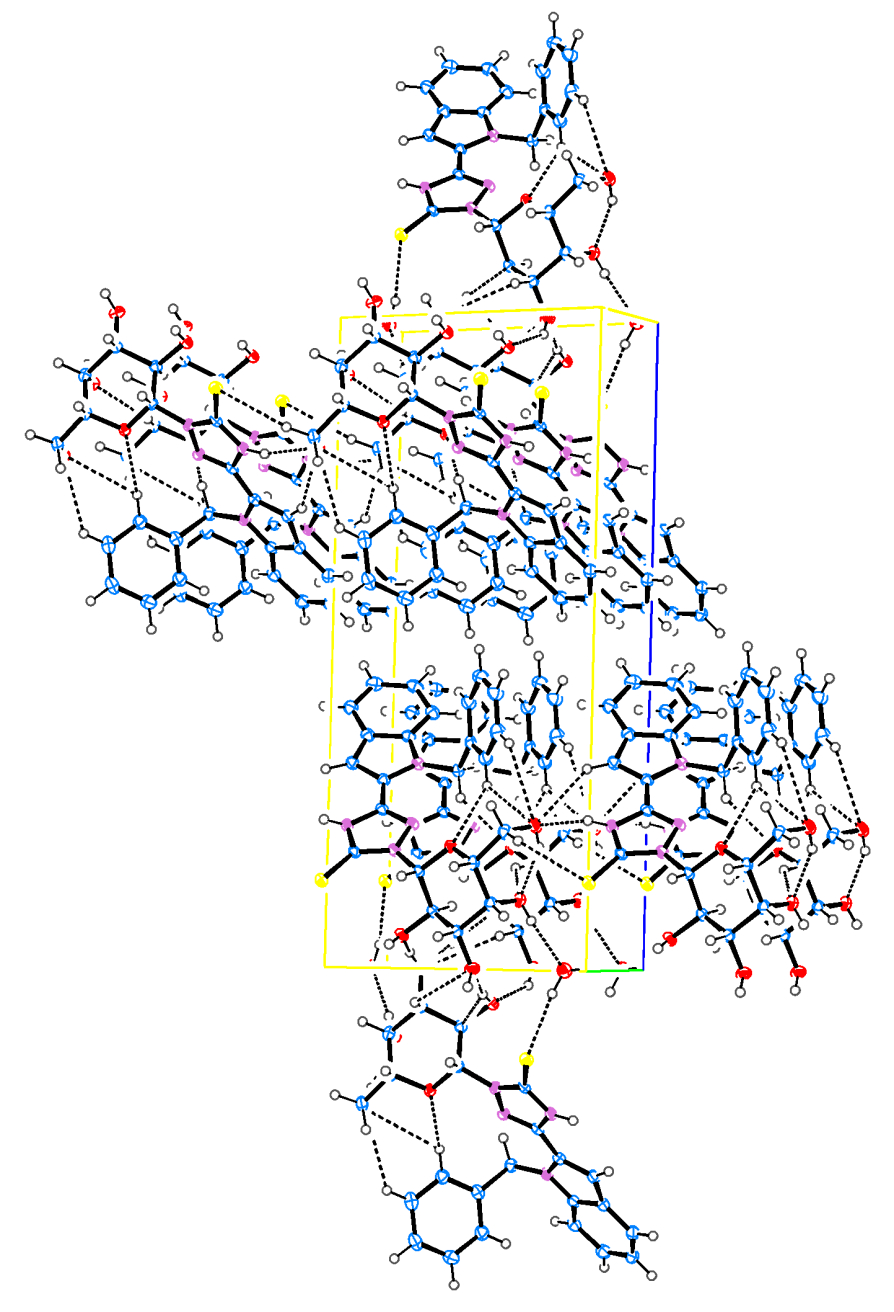
Packing diagram of **30**.

Single-crystal diffraction analysis of **30** (Figure 5, Figure 6 and Table 3) showed that the S(1)–C(17) bond length of 1.694 Å reflects double-bond character (similar to the bond lengths of S(1)–C(17) and S(2)–C(40) with 1.699(3) and 1.660(3) Å, respectively, of **29**) and suggests the thione form. O(5)–C(18) and O(5)–C(22) bond lengths are 1.411 and 1.439 Å, respectively (again similar to those found in **23** and **29**), which shows that, first, the compound still has the cyclic galactopyranose structure, and second, the sugar moiety is stable, even if the oxadiazole moiety has opened and the TSC formed has cyclized to form a triazole. The whole structure is nonplanar and the phenyl group still oriented perpendicular to the indole ring making torsion angles of C(8)–N(1)–C(7)–C(1) = 85.2° and C(15)–N(1)–C(7)–C(1) = −88.4°. On the other hand, the triazole ring is located in the plane of the indole ring making only small torsion angles of C(14)–C(15)–C(16)–N(4) = 3.6(4)° and N(1)–C(15)–C(16)–N(2) = 2.2(4)°. The molecular conformation is stabilized by an intramolecular hydrogen bond O(4)–H(4)···O(3) and a water molecule links two molecules in the crystal lattice through an intermolecular hydrogen bond O(6)–H(6P)···S(1).

**Table 3:** Crystal data, instrumental and refinement data for **30**.

Crystal data	

Empirical formula	C_23_H_24_N_4_O_5_S·H_2_O
Formula weight	486.55
Crystal size	0.5666 × 0.0262 × 0.0131 mm
Crystal description	Needle
Crystal color	Colorless
Crystal system	Monoclinic
Space group	*P*2_1_
Unit cell dimensions	*a* = 9.7950 Å; α = 90° *b* = 4.93845 Å; β = 90.813° *c* = 22.3598 Å; γ = 90°
Volume	1081.48 Å^3^
*Z*	2
Calculated density	1.491 Mg/m^3^
Absorption coefficient	1.770 mm^−1^
F(000)	510

Data collection	

Measurement device type	SuperNova, single source at offset, Atlas
Measurement method	w Scans
Temperature	123 K
Wavelength	1.54184 Å
Monochromator	Graphite
Theta range for data collection	3.95 to 72.89°
Index ranges	−11<=*h*<=11, −5<=*k*<=3, −25<=*l*<=27
Reflections collected/unique	3935/3005 [R(int) = 0.0286]
Reflections greater	I>2\s(I) 2785
Absorption correction	Analytical
Max. and min. transmission	1.00000 and 0.86469

Refinement	

Refinement method	Full-matrix least-squares on F2
Hydrogen treatment; Data/restraints/parameters	3005/1/327
Goodness-of-fit on F2	1.027
Final R indices [I>2sigma(I)]	R1 = 0.0378, wR2 = 0.0933
R indices (all data)	R1 = 0.0422, wR2 = 0.0959
Absolute structure parameter	0.00(2)
Largest diff. peak and hole	0.372 and −0.231 *e*·Å^−3^

## Conclusion

In conclusion, 2-*N*-(glycosyl)thiosemicarbazides of type **II** (from the four glycosylthiosemicarbazide structural isomers **I–IV** shown in Scheme 1) were synthesized from 3-*N*-(glycosyl)oxadiazolinethiones, which were accessed by new regioselective glycosylations. Additionally, 3-*N*-(glycosyl)oxadiazolinethiones may be prepared by a mild solvent-free thermal *S*→*N* migration of the glycosyl moiety in glycosylsulfanyloxadiazoles. (Benzylindolyl)glycosylsulfanyl-1,3,4-oxadiazoles could be thermally rearranged into the corresponding *N*-glycosides. These may either be converted into the corresponding (benzylindolyl)*-*2-*N*-(glycosyl)thiosemicarbazides (of type **II)** or into the galactosyl triazolinethione from the galactosyl oxadiazolinethione, as confirmed by X-ray single-crystal analysis and from further common structural analytical data.

## Supporting Information

Complete crystallographic data of the structural analysis of compounds **23, 29** and **30** have been deposited with the Cambridge Crystallographic Data Centre, CCDC 867245–867247. Copies of this information may be obtained free of charge from the Director, Cambridge Crystallographic Data Centre, 12 Union Road, Cambridge, CB2 1EZ, UK. (fax: +44-1223-336033, e-mail: deposit@ccdc.cam.ac.uk or via http://www.ccdc.cam.ac.uk).

File 1 Complete experimental section with full characterization data of all compounds.

File 2 Chemical information files (cif) of compounds **23, 29** and **30**.
