# Supplementary material for: A new synthetic access to 2-N-(glycosyl)thiosemicarbazides from 3-N-(glycosyl)oxadiazolinethiones and the regioselectivity of the glycosylation of their oxadiazolinethione precursors
Source: Beilstein J Org Chem. 2013 Jan 21;9:135–46. doi: 10.3762/bjoc.9.16 (PMC3566832; doi:10.3762/bjoc.9.16)
Supplement: File 1 — Complete experimental section with full characterization data of all compounds. [file Beilstein_J_Org_Chem-09-135-s001.pdf]

Supporting Information

for

A new synthetic access to 2-*N*-(glycosyl)thiosemicarbazides from 3-*N*-(glycosyl)oxadiazolinethiones and the regioselectivity of the glycosylation of their oxadiazolinethione precursors

El Sayed H. El Ashry[1,2*§], El Sayed H. El Tamany[3], Mohy El Din Abdel Fattah[3], Mohamed R. E. Aly[4], Ahmed T. A. Boraei[1,3] and Axel Duerkop[5]

Address: [1]HEJ Research Institute of Chemistry, International Center for Chemical and Biological Sciences, Karachi University, Karachi, Pakistan, [2]Chemistry Department, Faculty of Science, Alexandria University, Alexandria, Egypt, [3]Chemistry Department, Faculty of Science, Suez Canal University, Ismailia, Egypt, [4]Chemistry Department, Faculty of Applied Science, Port Said University, Port Said, Egypt and [5]Institute of Analytical Chemistry, Chemo and Biosensors, Universitätsstrasse 31, 93053 Regensburg, Germany

Email: El Sayed H. El Ashry - eelashry60@hotmail.com

*Corresponding author

§Tel: +203-4246601; fax: +203-4271360.

Complete experimental section with full characterizations of all compounds

General details

Melting points were determined with a Stuart melting-point apparatus (SMP10) in open capillaries and are uncorrected. Flash chromatography was performed on silica gel 60 (230–400 mesh ASTM). TLC was performed on Merck silica gel 60 F_{254} and spots were visualized by absorption of UV light and/or treatment with a solution of 10% H_2SO_4 in aqueous methanol followed by heating. IR spectra were recorded using KBr discs on a Bruker FT-IR IFS 48 spectrophotometer. ^{1}H NMR spectra were recorded on Avance Bruker NMR spectrometers at 300–500 MHz whereas ^{13}C NMR spectra were done on the same instruments at 75–125 MHz, with TMS as internal standard. Mass spectra were recorded on a Finnigan MAT312 and a Jeol JMS.600H for EIMS; HRMS spectra were recorded on a Thermo Finnigan MAT 95XP and a Jeol JMS HX110. Single crystals suitable for structural analysis were obtained by careful recrystallization from ethanol. X-ray crystallographic data were collected using an Oxford Diffraction SuperNova CCD diffractometer. The structures were solved by direct methods (*SIR97*) and refined by full-matrix anisotropic least squares (SHELXL97).

General methods for glycosylation of 5-(1*H*-indol-2-yl)-1,3,4-oxadiazole-2(3*H*)-thione (1)

Method a: A mixture of the appropriate glycosyl halide **2–4** (1.0 g, 2.4 mmol), **1** (0.5 g, 2.3 mmol) and basic alumina (0.5–1.0 g) in acetone (1.0 mL) was subjected to evaporation in vacuo. The resulting solid mixture was ground for 10 min, then suspended in CH_2Cl_2 or acetone (3.0 mL) and filtered, and the solid was thoroughly washed with the same solvent. The filtrate was evaporated in vacuo. The residue was crystallized from EtOH to afford the *S*-glycosides **5–7**.

Method b: A mixture of **1** (0.5 g, 2.3 mmol) and mercuric chloride (0.68 g, 2.5 mmol) in ethanol was stirred for 15 min. The precipitated chloromercuric salt was filtered, washed with EtOH and dried. A mixture of the chloromercuric salt (0.5 g, 1.1 mmol) and the appropriate glycosyl halide **2–4** (0.49 g, 1.2 mmol) in dry toluene (15 mL) were heated under reflux until

completion of the reaction as indicated by TLC. After being cooled to room temperature, the mixture was filtered and the filtrate was evaporated in vacuo. The residue was crystallized from EtOH to afford the *N*-glycosides **8–10**.

Method c: The appropriate glycosyl halide **2–4** (1.0 g, 2.4 mmol) was added in portions to a well-stirred mixture of **1** (0.5 g, 2.3 mmol) and Et_3N (1.0 mmol) in dry acetone (10 mL), and stirring was continued overnight. The mixture was filtered and washed thoroughly with acetone, and the filtrate was evaporated in vacuo. Flash chromatography of the residues using ethyl acetate/*n*-hexane (3:7) for glycosides **5–6**/**8–9** and $CHCl_3$/MeOH (9.5:0.5) for glycosides **7** and **10** affords the corresponding pure *S*- and *N*-glycosides.

Method d: The same as *Method c* except using K_2CO_3 instead of Et_3N.

General procedure for thermal conversion of 5–7 into 8–10

The *S*-glycoside **5–7** or a mixture of both *S*- and *N*-glycosides after removing the base and solvent (0.1 g, 0.18 mmol) was fused under atmospheric conditions, and the thermal conversion was monitored by TLC. After completion of the reaction (5–10 min), the fused mass was allowed to reach ambient temperature, and the resulting solid was ground and then crystallized from EtOH to afford the corresponding *N*-glycosides **8–10**.

2-(2,3,4,6-Tetra-*O*-acetyl-β-D-glucopyranosylsulfanyl)-5-(1*H*-indol-2-yl)-1,3,4-oxadiazole (5)

Yields: (0.743 g, 59% *Method a*), (0.768 g, 61% *Method c* [1]) and (0.605 g, 48% *Method d* [1]) as colorless crystals. Mp 215–218 °C [1]. TLC (5.5:4.5 *n*-hexane/ethyl acetate): R_f 0.35. IR (KBr): ν_{max}/cm^{-1} 3249 (NH_{Indol}), 1748.7 (C=O), 1608 (C=N). ^1H NMR (300 MHz, $CDCl_3$): δ 1.99, 2.02, 2.03, 2.06 (4s, 12 H, 4 CH_3CO), 3.92-3.95 (m, 1 H, H-5_{Glc}), 4.14 (dd, J_{gem} 12.5, $J_{5,6}$ 1.9 Hz, 1 H, H-6_{Glc}), 4.27 (dd, J_{gem} 12.5, $J_{5,6'}$ 4.6 Hz, 1 H, H-$6'_{Glc}$), 5.17 (dd, $J_{3,4}$ 9.3, $J_{4,5}$ 9.5 Hz, 1 H, H-4_{Glc}), 5.24 (dd, $J_{1,2}$ 10.3, $J_{2,3}$ 9.3 Hz, 1 H, H-2_{Glc}), 5.42 (dd, $J_{2,3} = J_{3,4}$ 9.3 Hz, 1

H, H-3$_{Glc}$), 5.55 (d, $J_{1,2}$ 10.3 Hz, 1 H, H-1$_{Glc}$), 7.14-7.24 (m, H-3$_{Indol}$, 2 H, H-5$_{Indol}$), 7.32 (dd, $J_{5,6}$ 7.2, $J_{6,7}$ 8.3 Hz, 1 H, H-6$_{Indol}$), 7.58 (d, $J_{6,7}$ 8.3 Hz, 1 H, H-7$_{Indol}$), 7.69 (d, $J_{4,5}$ 8.0 Hz, 1 H, H-4$_{Indol}$), 9.95 (br. s, 1 H, NH$_{Indol}$). ^{13}C NMR (75 MHz, CDCl$_3$): δ 20.54, 20.57, 20.62 (4 *C*H$_3$CO), 61.58 (C-6$_{Glc}$), 67.84 (C-4$_{Glc}$), 69.9 (C-2$_{Glc}$), 73.63 (C-3$_{Glc}$), 76.58 (C-5$_{Glc}$), 83.43 (C-1$_{Glc}$), 106.66 (C-3$_{Indol}$), 111.96 (C-7$_{Indol}$), 120.40 (C-2$_{Indol}$), 121.19 (C-5$_{Indol}$), 122.01 (C-4$_{Indol}$), 125.34 (C-6$_{Indol}$), 127.70 (C-3a$_{Indol}$), 137.60 (C-7a$_{Indol}$), 160.20 (C-5$_{Oxad.}$), 161.47 (C-2$_{Oxad.}$), 169.34, 169.47, 170.07, 170.6 (4 CH$_3$*C*O). HRMS (EI): *m*/*z* calcd for $C_{24}H_{25}N_3O_{10}S$ [M$^+$] 547.1260, found 547.1259.

2-(2,3,4,6-Tetra-*O*-acetyl-β-D-galactopyranosylsulfanyl)-5-(1*H*-indol-2-yl)-1,3,4-oxadiazole (6)

Yields: (0.793 g, 63% *Method a*), (0.894 g, 71% *Method c* [1]) and (0.696 g, 58% *Method d* [1]) as colorless crystals. Mp 205–207 °C [1]. TLC (5.5:4.5 *n*-hexane/ethyl acetate): R_f 0.36. IR (KBr): ν_{max}/cm^{-1} 3378 (NH$_{Indol}$), 1748 (C=O), 1630 (C=N). ^{1}H NMR (500 MHz, CDCl$_3$,): δ 1.97, 1.99, 2.09, 2.13 (4s, 12 H, 4 *C*H$_3$CO), 4.08-4.13 (m, 3 H, H-5$_{Gal}$, H-6$_{Gal}$, H-6$'_{Gal}$), 5.15 (dd, $J_{2,3}$ 9.4, $J_{3,4}$ 3.3 Hz, 1 H, H-3$_{Gal}$), 5.45-5.48 (m, 3 H, H-1$_{Gal}$, H-2$_{Gal}$, H-4$_{Gal}$), 7.16-7.19 (m, 2 H, H-3$_{Indol}$, H-5$_{Indol}$), 7.33 (dd, $J_{5,6}$ 7.4, $J_{6,7}$ 8.2 Hz, 1 H, H-6$_{Indol}$), 7.47 (d, $J_{6,7}$ 8.2 Hz, 1 H, H-7$_{Indol}$), 7.68 (d, $J_{4,5}$ 8.0 Hz, 1 H, H-4$_{Indol}$), 9.20 (br. s, 1 H, NH$_{Indol}$). ^{13}C NMR (125 MHz, CDCl$_3$): δ 20.55, 20.67 (4 *C*H$_3$CO), 61.2 (C-6$_{Gal}$), 67.00, 67.20 (C-4$_{Gal}$, C-2$_{Gal}$), 71.60 (C-3$_{Gal}$), 75.30 (C-5$_{Gal}$), 84.00 (C-1$_{Gal}$), 106.70 (C-3$_{Indol}$), 112.10 (C-7$_{Indol}$), 120.40 (C-2$_{Indol}$), 121.20 (C-5$_{Indol}$), 121.90 (C-4$_{Indol}$), 125.30 (C-6$_{Indol}$), 127.60 (C-3a$_{Indol}$), 137.70 (C-7a$_{Indol}$), 160.40 (C-5$_{Oxad.}$), 161.50 (C-2$_{Oxad.}$), 169.70, 169.90, 170.10, 170.30 (4 CH$_3$*C*O). HRMS (EI): *m*/*z* calcd for $C_{24}H_{25}N_3O_{10}S$ [M$^+$] 547.1260, found 547.1259.

2-(2-Acetamido-2-deoxy-3,4,6-tri-*O*-acetyl-β-D-glucopyranosylsulfanyl)-5-(1*H*-indol-2-yl)-1,3,4-oxadiazole (7)

Yields: (0.654 g, 52% *Method a*), (0.666 g, 53% *Method c* [1]) and (0.528 g, 42% *Method d* [1]) as a yellowish white powder. Mp 250–251 °C [1]. TLC (9.5:0.5 $CHCl_3$/MeOH): R_f 0.50. IR (KBr): ν_{max}/cm^{-1} 3318 (NH_{Indol}), 1748 (C=O), 1661 (*Amide I*), 1617 (C=N), 1529 (*Amide II*). ^{1}H NMR (300 MHz, DMSO-d_6): δ 1.83 (s, 3 H, NHCH_3CO), 1.94, 1.98, 2.00 (3s, 9 H, 3 CH_3CO), 3.98-4.18 (m, 4 H, H-2_{GlcNAc}, H-5_{GlcNAc}, H-6_{GlcNAc}, H-$6'_{GlcNAc}$), 4.92 (dd, $J_{3,4} \approx J_{4,5}$ 9.6 Hz, 1H, H-4_{GlcNAc}), 5.22 (dd, $J_{2,3}$ 9.7, $J_{3,4}$ 9.6 Hz, 1 H, H-3_{GlcNAc}), 5.56 (d, $J_{1,2}$ 10.6 Hz, 1H, H-1_{GlcNAc}), 7.10 (dd, $J_{4,5}$ 8.0, $J_{5,6}$ 7.5 Hz, 1 H, H-5_{Indol}), 7.23-7.29 (m, 2 H, H-3_{Indol}, H-6_{Indol}), 7.47 (d, $J_{6,7}$ 8.3 Hz, 1 H, H-7_{Indol}), 7.67 (d, $J_{4,5}$ 8.0 Hz, 1H, H-4_{Indol}), 8.27 (d, *J* 9.21 Hz, 1 H, N*H*Ac), 12.26 (br. s, 1 H, NH_{Indol}). ^{13}C NMR (75 MHz, DMSO-d_6): δ 20.10, 20.20, 20.30, 22.50 (4 COCH_3), 52.10 (C-2_{GlcNAc}), 61.70 (C-6_{GlcNAc}), 68.30 (C-4_{GlcNAc}), 72.80 (C-3_{GlcNAc}), 75.20 (C-5_{GlcNAc}), 83.90 (C-1_{GlcNAc}), 105.40 (C-3_{Indol}), 112.30 (C-7_{Indol}), 120.40 (C-2_{Indol}), 120.50 (C-5_{Indol}), 121.40 (C-4_{Indol}), 124.40 (C-6_{Indol}), 127.20 (C-$3a_{Indol}$), 137.80 (C-$7a_{Indol}$), 159.70 (C-$5_{Oxad.}$), 161.10 (C-$2_{Oxad.}$), 169.20, 169.60, 169.70, 169.80 (4 *C*OCH_3). HRMS (ESI): *m*/*z* calcd for $C_{24}H_{27}N_4O_9S$ [M + H^+] 547.1498, found 547.1525.

3-(2,3,4,6-Tetra-*O*-acetyl-β-D-glucopyranosyl)-5-(1*H*-indol-2-yl)-1,3,4-oxadiazole-2(3*H*)-thione (8)

Yields: (0.362 g, 60% *Method b*), (0.239 g, 19% *Method c* [1]), (0.403 g, 32% *Method d* [1]) and (0.068 g, 68% *Thermal rearrangement*) as a white powder. Mp 276–278 °C [1]. TLC (5.5:4.5 *n*-hexane/ethyl acetate): R_f 0.55. IR (KBr): ν_{max}/cm^{-1} 3295 (NH_{Indol}), 1746.5 (C=O), 1629 (C=N). ^{1}H NMR (300 MHz, $CDCl_3$): δ 1.95, 2.02, 2.06, 2.08 (4s, 12 H, 4 CH_3CO), 3.96-4.01 (m, 1 H, H-5_{Glc}), 4.14 (dd, J_{gem} 12.5, $J_{5,6}$ 2.0 Hz, 1 H, H-6_{Glc}), 4.33 (dd, J_{gem} 12.5, $J_{5,6'}$ 5.0 Hz, 1 H, H-$6'_{Glc}$), 5.22 (dd, $J_{3,4}$ 9.5, $J_{4,5}$ 9.9 Hz, 1 H, H-4_{Glc}), 5.40 (dd, $J_{2,3} = J_{3,4}$ 9.5 Hz, 1

H, H-3$_{Glc}$), 5.63 (dd, $J_{1,2}$ 9.3, $J_{2,3}$ 9.5 Hz, 1 H, H-2$_{Glc}$), 5.92 (d, $J_{1,2}$ 9.3 Hz, 1 H, H-1$_{Glc}$), 7.17 (dd, $J_{4,5}$ 8.0, $J_{5,6}$ 7.5 Hz, 1 H, H-5$_{Indol}$), 7.24 (s, 1 H, H-3$_{Indol}$), 7.34 (dd, $J_{5,6}$ 7.5, $J_{6,7}$ 8.3 Hz, 1 H, H-6$_{Indol}$), 7.42 (d, $J_{6,7}$ 8.3 Hz, 1 H, H-7$_{Indol}$), 7.67 (d, $J_{4,5}$ 8.0 Hz, 1 H, H-4$_{Indol}$), 8.87 (br. s, 1 H, NH$_{Indol}$). ^{13}C NMR (75 MHz, CDCl$_3$): δ 20.53, 20.69 (4 *C*H$_3$CO), 61.61 (C-6$_{Glc}$), 67.70 (C-4$_{Glc}$), 69.20 (C-2$_{Glc}$), 73.20 (C-3$_{Glc}$), 74.80 (C-5$_{Glc}$), 83.10 (C-1$_{Glc}$), 108.34 (C-3$_{Indol}$), 111.70 (C-7$_{Indol}$), 119.00 (C-2$_{Indol}$), 121.50 (C-5$_{Indol}$), 122.30 (C-4$_{Indol}$), 126.00 (C-6$_{Indol}$), 127.50 (C-3a$_{Indol}$), 137.70 (C-7a$_{Indol}$), 155.00 (C-5$_{Oxad.}$), 169.10, 169.40, 170.00, 170.50 (4 CH$_3$*C*O), 177.40 (C=S). HRMS (EI): *m/z* calcd for $C_{24}H_{25}N_3O_{10}S$ [M$^+$] 547.1260, found 547.1259.

3-(2,3,4,6-Tetra-*O*-acetyl-β-D-galactopyranosyl)-5-(1*H*-indol-2-yl)-1,3,4-oxadiazole-2(3*H*)-thione (9)

Yields: (0.344 g, 57% *Method b*), (0.151 g, 12% *Method c* [1]), (0.315 g, 25% *Method d* [1]) *and* (0.06 g, 60% *Thermal rearrangement*) as colorless crystals. Mp 231–233 °C [1]. TLC (5.5:4.5 *n*-hexane/ethyl acetate): R_f 0.53. ^{1}H NMR (300 MHz, CDCl$_3$): δ 1.96, 2.00, 2.05, 2.24 (4s, 12 H, 4 CH$_3$CO), 4.19-4.20 (m, 3 H, H-5$_{Gal}$, H-6$_{Gal}$, H-6$'_{Gal}$), 5.24 (dd, $J_{2,3}$ 10.2, $J_{3,4}$ 3.4 Hz, 1 H, H-3$_{Gal}$), 5.51 (d, $J_{3,4} \approx J_{4,5}$ 3.4 Hz, 1 H, H-4$_{Gal}$), 5.74 (dd, $J_{1,2}$ 9.2, $J_{2,3}$ 9.9 Hz, 1 H, H-2$_{Gal,}$), 5.90 (d, $J_{1,2}$ 9.2 Hz, 1 H, H-1$_{Gal}$), 7.17 (dd, $J_{4,5}$ 8.0, $J_{5,6}$ 7.3 Hz, 1 H, H-5$_{Indol}$), 7.23 (s, 1 H, H-3$_{Indol}$), 7.34 (dd, $J_{5,6}$ 7.3, $J_{6,7}$ 8.3 Hz, 1H, H-6$_{Indol}$), 7.42 (d, $J_{6,7}$ 8.3 Hz, 1 H, H-7$_{Indol}$), 7.68 (d, $J_{4,5}$ 8.0 Hz, 1 H, H-4$_{Indol}$), 8.87 (br. s, 1 H, NH$_{Indol}$). ^{13}C NMR (75 MHz, CDCl$_3$): δ 20.48, 20.61, 20.64, 20.70 (4 *C*H$_3$CO), 61.1 (C-6$_{Gal}$), 66.90 (C-2$_{Gal}$, C-4$_{Gal}$), 71.3 (C-3$_{Gal}$), 73.80 (C-5$_{Gal}$), 83.6 (C-1$_{Gal}$), 108.3 (C-3$_{Indol}$), 111.7 (C-7$_{Indol}$), 119.1 (C-2$_{Indol}$), 121.5 (C-5$_{Indol}$), 122.3 (C-4$_{Indol}$), 126.0 (C-6$_{Indol}$), 127.5 (C-3a$_{Indol}$), 137.6 (C-7a$_{Indol}$), 154.5 (C-5$_{Oxad.}$), 169.2, 169.8, 170.1, 170.3 (4 CH$_3$*C*O), 177.3 (C=S). HRMS (EI): *m/z* calcd for $C_{24}H_{25}N_3O_{10}S$ [M$^+$] 547.1260, found 547.1259.

3-(2-Acetamido-2-deoxy-3,4,6-tri-*O*-acetyl-β-D-glucopyranosyl)-5-(1*H*-indol-2-yl)-1,3,4-oxadiazole-2(3*H*)-thione (10)

Yields: (0.289 g, 48% *Method b*), (0.289 g, 23% *Method c* [1]), (0.44 g, 35% *Method d* [1]) and (0.09 g, 90% *Thermal rearrangement*). Mp 292–294 °C [1]. TLC (9.5:0.5 $CHCl_3$/MeOH): R_f 0.80. IR (KBr): ν_{max}/cm^{-1} 3293 (NH_{Indol}), 1745 (C=O), 1668 (*Amide I*), 1617 (C=N), 1531 (*Amide II*), ^1H NMR (300 MHz, DMSO-d_6): δ 1.67, 1.95, 2.00, 2.01 (4s, 12 H, 4 $COCH_3$), 4.08 (m, 1 H, H-5_{GlcNAc}), 4.24 (m, 2 H, H-6_{GlcNAc}, H-$6'_{GlcNAc}$), 4.36 (dd, $J_{1,2}$ 9.5, $J_{2,3}$ 9.9 Hz, 1 H, H-2_{GlcNAc}), 4.96 (dd, $J_{3,4}$ 9.6, $J_{4,5}$ 9.8 Hz, 1 H, H-4_{GlcNAc}), 5.50 (dd, $J_{2,3}$ 9.9, $J_{1,2}$ 9.5 Hz, 1 H, H-3_{GlcNAc}), 6.10 (d, $J_{1,2}$ 9.5 Hz, 1 H, H-1_{GlcNAc}), 7.11 (dd, $J_{4,5}$ 8.0, $J_{5,6}$ 7.5 Hz, 1 H, H-5_{Indol}), 7.26-7.31 (m, 2 H, H-3_{Indol}, H-6_{Indol}), 7.47 (d, $J_{6,7}$ 8.2 Hz, 1 H, H-7_{Indol}), 7.68 (d, $J_{4,5}$ 8.0 Hz, 1 H, H-4_{Indol}), 8.07 (d, *J* 8.7 Hz, 1 H, N*H*Ac), 12.23 (br. s, 1 H, NH_{Indol}). ^{13}C NMR (75 MHz, DMSO-d_6): δ 20.29, 20.34, 20.50, 22.50 (4 CO*C*H_3), 51.80 (C-2_{GlcNAc}), 61.50 (C-6_{GlcNAc}), 67.90 (C-4_{GlcNAc}), 73.20 (C-5_{GlcNAc}), 83.20 (C-1_{GlcNAc}), 106.50 (C-3_{Indol}), 112.40 (C-7_{Indol}), 119.10 (C-2_{Indol}), 120.60 (C-5_{Indol}), 121.70 (C-4_{Indol}), 124.90 (C-6_{Indol}), 126.90 (C-$3a_{Indol}$), 138.10 (C-$7a_{Indol}$), 154.30 (C-$5_{Oxad.}$), 169.30, 169.40, 169.80, 169.90 (4 *C*OCH_3), 176.10 (C=S). HRMS (ESI): *m/z* calcd for $C_{24}H_{27}N_4O_9S$ [M + H^+] 547.1498, found 547.1500.

General procedure for the synthesis of 2-*N*-(glycopyranosyl)thiosemicarbazides 11–13

A mixture of the appropriate glycoside **8–10** (0.1 g, 0.18 mmol) in methanol (10 mL) and ammonia solution (32%, 5 mL) was stirred at 0 °C for 1 h and then allowed to reach ambient temperature, and stirring was continued for a further 6–8 h. The solvent was evaporated in vacuo and the residue was purified either by crystallization from ethanol/n-hexane or by column chromatography (ethyl acetate/MeOH/$CHCl_3$, 4:3:3).

2-(β-D-Glucopyranosyl)-1-(1*H*-indol-2-ylcarbonyl)thiosemicarbazide (11)

Yield: (0.044 g, 62%) as yellowish white crystals. Mp 212–214 °C [1]. TLC (4:3:3 ethyl acetate/MeOH/$CHCl_3$): R_f 0.50. IR (KBr): ν_{max}/cm^{-1} 3375-3114 (NH_{Indol}, 4 OH_{Glc}, NH_2), 1629 (*Amide I*, C=N), 1544 (*Amide II*). ^1H NMR (300 MHz, acetone-d_6 + D_2O): δ 3.19-3.80 (m, 6 H, H-2_{Glc}, H-3_{Glc}, H-4_{Glc}, H-5_{Glc}, H-6_{Glc}, H-$6'_{Glc}$), 6.55 (d, $J_{1,2}$ 8.5 Hz, 1 H, H-1_{Glc}), 7.07 (dd, $J_{4,5}$ 8.0, $J_{5,6}$ 7.5 Hz, 1 H, H-5_{Indol}), 7.25 (dd, $J_{5,6}$ 7.5, $J_{6,7}$ 8.3 Hz, 1 H, H-6_{Indol}), 7.42 (s, 1 H, H-3_{Indol}), 7.53 (d, $J_{6,7}$ 8.3 Hz, 1 H, H-7_{Indol}), 7.64 (d, $J_{4,5}$ 8.0 Hz, 1 H, H-4_{Indol}). ^{13}C NMR (75 MHz, DMSO-d_6): δ 60.7 (C-6_{Glc}), 69.7, 70.4, 75.7, 78.8 (C-2_{Glc}, C-3_{Glc}, C-4_{Glc}, C-5_{Glc}), 86.7 (C-1_{Glc}), 106.0 (C-3_{Indol}), 112.3 (C-7_{Indol}), 120.0 (C-5_{Indol}), 121.8 (C-4_{Indol}), 124.3 (C-6_{Indol}), 126.7 (C-$3a_{Indol}$), 128.01 (C-2_{Indol}), 136.86 (C-$7a_{Indol}$), 162.90 (C=O), 184.50 (C=S). HRMS (FAB): *m/z* calcd for $C_{16}H_{21}N_4O_6S$ [M + H^+] 397.1181, found 397.1175.

2-(β-D-Galactopyranosyl)-1-(1*H*-indol-2-ylcarbonyl)thiosemicarbazide (12)

Yield: (0.051 g, 71%) as colorless crystals. Mp 237–239 °C. TLC (4:3:3 ethyl acetate/MeOH/$CHCl_3$): R_f 0.51. IR (KBr): ν_{max}/cm^{-1} 3406-3195 (NH, OH and $NH_{2\ str}$), 1639 (*Amide I*), 1540 (*Amide II*). ^1H NMR (300 MHz, acetone-d_6 + D_2O,): δ 3.57-3.77 (m, 5 H, H-2_{Gal}, H-3_{Gal}, H-5_{Gal}, H-6_{Gal}, H-$6'_{Gal}$), 3.91 (d, $J_{3,4} \approx J_{4,5}$ 2.7 Hz, 1H, H-4_{Gal}), 6.46 (d, $J_{1,2}$ 8.5 Hz, 1H, H-1_{Gal}), 7.08 (dd, $J_{4,5}$ 8.0, $J_{5,6}$ 7.4 Hz, 1 H, H-5_{Indol}), 7.26 (dd, $J_{5,6}$ 7.4, $J_{6,7}$ 8.0 Hz, 1 H, H-6_{Indol}), 7.43 (s, 1 H, H-3_{Indol}), 7.53 (d, $J_{6,7}$ = 8.0 Hz, 1 H, H-7_{Indol}), 7.65 (d, $J_{4,5}$ 8.0 Hz, 1 H, H-4_{Indol}). ^{13}C NMR (100 MHz, DMSO-d_6): δ 60.1 (C-6_{Gal}), 67.61, 67.69, 72.85, 77.68 (C-2_{Gal}, C-3_{Gal}, C-4_{Gal}, C-5_{Gal}), 87.8 (C-1_{Gal}), 106.18 (C-3_{Indol}), 112.3 (C-7_{Indol}), 120.0 (C-5_{Indol}), 121.80 (C-4_{Indol}), 124.20 (C-6_{Indol}), 126.73, 128.08 (C-2_{Indol}, C-$3a_{Indol}$), 136.80 (C-$7a_{Indol}$), 162.80 (C=O), 184.50 (C=S). HRMS (FAB): *m/z* calcd for $C_{16}H_{21}N_4O_6S$ [M + H^+] 397.1181, found 397.1175.

2-(2-Acetamido-2-deoxy-β-D-glucopyranosyl)-1-(1*H*-indol-2-ylcarbonyl)thiosemicarbazide (13)

Yield (0.052 g, 65%) as yellowish white crystals. Mp 193–195 ºC. TLC (4:3:3 ethyl acetate/MeOH/$CHCl_3$): R_f 0.50, IR (KBr): ν_{max}/cm^{-1} 3291 (NH, OH and $NH_{2\ str}$), 1663, 1622 (2 Amide I and C=N), 1533 (amide II). ^1H NMR (300 MHz, acetone-d_6 + D_2O): δ 2.07 (s, 3 H, $COCH_3$), 3.13-3.88 (m, 6 H, H-2_{GlcNAc}, H-3_{GlcNAc}, H-4_{GlcNAc}, H-5_{GlcNAc}, H-6_{GlcNAc}, H-$6'_{GlcNAc}$), 6.53 (d, $J_{1,2}$ 9.0 Hz, 1 H, H-1_{GlcNAc}), 7.07 (dd, $J_{4,5}$ 8.0, $J_{5,6}$ 7.6 Hz, 1 H, H-5_{Indol}), 7.23 (dd, $J_{5,6}$ 7.6, $J_{6,7}$ 8.3 Hz, 1 H, H-6_{Indol}), 7.30 (s, 1 H, H-3_{Indol}), 7.52 (d, $J_{6,7}$ 8.3 Hz, 1 H, H-7_{Indol}), 7.67 (d, $J_{4,5}$ 8.0 Hz, 1 H, H-4_{Indol}). ^{13}C NMR (75 MHz, DMSO-d_6): δ 22.60 (COCH_3), 53.10 (C-2_{GlcNAc}), 61.20 (C-6_{GlcNAc}), 70.70, 73.80, 79.00 (C-3_{GlcNAc}, C-4_{GlcNAc}, C-5_{GlcNAc}), 86.80 (C-1_{GlcNAc}), 103.10 (C-3_{Indol}), 112.30 (C-7_{Indol}), 120.00 (C-5_{Indol}), 121.60 (C-4_{Indol}), 123.70 (C-6_{Indol}), 126.90, 129.80 (C-2_{Indol}, C-$3a_{Indol}$), 136.60 (C-$7a_{Indol}$), 159.40 (C=O), 172.50 (NH*C*OCH_3), 183.10 (C=S). HRMS (ESI): *m*/*z* calcd for $C_{18}H_{24}N_5O_6S$ [M + H^+] 438.1447, found 438.1510.

General procedure for the ammonia-mediated hydrolysis of *S*-glycosides (5–7)

The appropriate *S*-glycoside **5**–**7** (0.547 g, 1.0 mmol) in MeOH (10 mL) was treated with NH_4OH (32%, 5 mL), then worked up as described for compounds **11–13**. The residue was purified by crystallization from *n*-hexane/EtOH to afford **20** as colorless crystals in yields: 0.128 g (64% from **5**), 0.110 g (55% from **6**) and 0.084 g (42% from **7**).

5-(1*H*-Indol-2-yl)-1,3,4-oxadiazol-2(3*H*)-one (20)

Mp 273–275 °C, Lit. [2] 102 °C, Lit. [1] 271–273 °C, Lit. [3] 285 °C. TLC (9.5:0.5 $CHCl_3$/MeOH): R_f 0.54. ^1H NMR (300 MHz, DMSO-d_6): δ 7.00 (d, *J* 1.4 Hz, 1 H, H-3_{Indol}), 7.06 (dd, $J_{4,5}$ 7.9, $J_{5,6}$ 7.3 Hz, 1 H, H-5_{Indol}), 7.22 (dd, $J_{5,6}$ 7.3, $J_{6,7}$ 8.2 Hz, 1 H, H-6_{Indol}), 7.42

(d, $J_{6,7}$ 8.2 Hz, 1 H, H-7$_{Indol}$), 7.62 (d, $J_{4,5}$ 7.9 Hz, 1 H, H-4$_{Indol}$), 11.98 (br. s, 1 H, NH$_{Indol}$), 12.60 (br. s, 1 H, NH$_{Oxad.}$). ^{13}C NMR (75 MHz, DMSO-d_6): δ 103.8 (C-3$_{Indol}$), 111.99 (C-7$_{Indol}$), 120.10 (C-5$_{Indol}$), 121.19 (C-4$_{Indol}$), 121.60 (C-2$_{Indol}$), 123.86 (C-6$_{Indol}$), 127.00 (C-3a$_{Indol}$), 137.00 (C-7a$_{Indol}$), 149.50 (C-5$_{Oxad.}$), 154.00 (C=O). HRMS (EI): *m*/*z* calcd for $C_{10}H_7N_3O_2$ 201.0538, found 201.0541.

Methyl 2-(1*H*-indol-2-ylcarbonyl)hydrazinecarboxylate (22)

Compound **21** (0.2 g, 1.1 mmol) was heated under reflux with methyl chloroformate (1.0 mmol) in ethanol (5 mL) until completion of the reaction (8 h) as indicated by TLC, then allowed to reach ambient temperature. The precipitate formed was filtered, dried and recrystallized from ethanol to afford **22** in a yield: (0.228 g, 86%) as colorless crystals. Mp 247–248 °C. TLC (9.5:0.5 $CHCl_3$/MeOH): R_f 0.54. ^{1}H NMR (300 MHz, DMSO-d_6): δ 3.63 (s, 3 H, CH_3), 7.04 (dd, $J_{4,5}$ 7.96, $J_{5,6}$ 7.4 Hz, 1 H, H-5$_{Indol}$), 7.17-7.19 (m, 2 H, H-3$_{Indol}$, H-6$_{Indol}$), 7.43 (d, $J_{6,7}$ 8.2 Hz, 1 H, H-7$_{Indol}$), 7.62 (d, $J_{4,5}$ 7.96 Hz, 1H, H-4), 9.25 (br.s, 1 H, NH), 10.30 (br.s, 1 H, NH), 11.67 (br. s, 1H, NH$_{Indol}$). ^{13}C NMR (DMSO-d_6, 75 MHz): δ 51.97 (CH_3), 103.27 (C-3), 112.29 (C-7), 119.86 (C-5), 120.65 (C-4), 123.65 (C-6), 126.89 (C-3a), 129.34 (C-2), 136.57 (C-7a), 156.84, 160.98 (2 CO). HRMS (EI): *m*/*z* calcd for $C_{11}H_{11}N_3O_3$ [M^+] 233.0800, found 233.0800.

A suspension of **22 (**0.23 g, 1.0 mmol**)** in diphenyl ether (10 mL) was heated under reflux for 3 h then allowed to reach ambient temperature and was then diluted with *n*-hexane. The precipitate was filtered, washed thoroughly with *n*-hexane, dried and recrystallized from ethanol/*n*-hexane to afford **20** (0.158 g, 80%) as colorless crystals. It has physical constants and spectra identical with those obtained above.

General procedure for synthesis of 23–28

Benzyl bromide (0.19 g, 1.1 mmol) was added in portions to a well-stirred mixture of the appropriate glycoside **5–10** (0.54 g, 0.99 mmol) and K_2CO_3 (0.15 g, 1.1 mmol) in dry acetone (10 mL), and stirring was continued overnight. The mixture was filtered and washed thoroughly with acetone, and the solvent was evaporated in vacuo. The residue was purified by flash chromatography (*n*-hexane/ethyl acetate, 2:3).

2-(2,3,4,6-Tetra-*O*-acetyl-β-D-glucopyranosylsulfanyl)-5-(1-benzyl-1*H*-indol-2-yl)-1,3,4-oxadiazole (23)

Yield: (0.251 g, 40%) as colorless sticklike crystals. Mp 152–153 °C. TLC (2:3 *n*-hexane/ethyl acetate): R_f 0.38. ^{1}H NMR (400 MHz, $CDCl_3$): δ 1.98, 2.01, 2.02, 2.05 (4s, 12 H, 4 CH_3CO), 3.83 (m, 1 H, H-5_{Glc}), 4.10 (dd, J_{gem} 12.6, $J_{5,6}$ 1.8 Hz, 1 H, H-6_{Glc}), 4.24 (dd, J_{gem} 12.6, $J_{5,6'}$ 4.8 Hz, 1 H, H-$6'_{Glc}$), 5.14 (dd, $J_{3,4}$ 9.2, $J_{4,5}$ 9.6 Hz, 1 H, H-4_{Glc}), 5.21 (dd, $J_{1,2}$ 10.4, $J_{2,3}$ 9.6 Hz, 1 H, H-2_{Glc}), 5.29 (dd, $J_{2,3}$ 9.6, $J_{3,4}$ 9.2 Hz, 1 H, H-3_{Glc}), 5.49 (d, $J_{1,2}$ 10.4 Hz, 1 H, H-1_{Glc}), 5.97 (s, 2 H, NCH_2), 7.04 (d, *J* 6.8 Hz, 2 H, $2H_{Ph}$), 7.15-7.24 (m, 4 H, H-5_{Indol}, $3H_{Ph}$), 7.26 (s, 1 H, H-3_{Indol}), 7.29 (dd, $J_{5,6}$ 6.8, $J_{6,7}$ 8.4 Hz, 1 H, H-6_{Indol}), 7.35 (d, $J_{6,7}$ 8.4 Hz, 1 H, H-7_{Indol}), 7.70 (d, $J_{4,5}$ 8.0 Hz, 1 H, H-4_{Indol}). ^{13}C NMR (100 MHz, $CDCl_3$): δ 20.5, 20.6 (4 *C*H_3CO), 48.4 (NCH_2), 61.60 (C-6_{Glc}), 67.80 (C-4_{Glc}), 69.80 (C-2_{Glc}), 73.60 (C-3_{Glc}), 76.60 (C-5_{Glc}), 83.40 (C-1_{Glc}), 107.90 (C-3_{Indol}), 110.90 (C-7_{Indol}), 121.20 (C-5_{Indol}), 122.20 (C-4_{Indol}), 125.20 (C-6_{Indol}), 126.40 (C-2_{indol}, $2C_{Ph}$), 127.06, 127.30 (C-$3a_{Indol}$, C_{Ph}), 128.60 ($2C_{Ph}$), 137.40 (C-$7a_{Indol}$), 139.50 (C_{Ph}), 160.03 (C-$5_{Oxad.}$), 161.20 (C-$2_{Oxad.}$), 169.30, 169.40, 169.90, 170.60 (4 CH_3*C*O_{Glc}). HRMS (EI): *m*/*z* calcd for $C_{31}H_{31}N_3O_{10}S$ [M^+] 637.1730, found 637.1739.

2-(2,3,4,6-Tetra-*O*-acetyl-β-D-galactopyranosylsulfanyl)-5-(1-benzyl-1*H*-indol-2-yl)-1,3,4-oxadiazole (24)

Yield: (0.270 g, 43%) as a yellowish white powder. Mp 91–93 °C, TLC (3:2 *n*-hexane/ethyl acetate): R_f 0.37. ^{1}H NMR (400 MHz, $CDCl_3$): δ 1.93, 1.99, 2.06, 2.13 (4s, 12 H, 4 CH_3CO), 4.04-4.15 (m, 3 H, H-5_{Gal}, H-6_{Gal}, H-$6'_{Gal}$), 5.13 (dd, $J_{2,3}$ 9.6, $J_{3,4}$, 3.2 Hz, 1 H, H-3_{Gal}), 5.40 (dd, $J_{1,2}$ 10.0, $J_{2,3}$ 9.6 Hz, 1 H, H-2_{Gal}), 5.45-5.48 (m, 2 H, H-4_{Gal}, H-1_{Gal}), 5.97 (s, 2 H, CH_2Ph), 7.04 (d, J 6.8 Hz, 2 H, Ph), 7.15-7.21 (m, 4 H, H-5_{Indol}, Ph), 7.27-7.31 (m, 2 H, H-3_{Indol}, H-6_{Indol}), 7.36 (d, $J_{6,7}$ 8.4 Hz, 1H, H-7_{Indol}), 7.70 (d, $J_{4,5}$ 8.0 Hz, 1 H, H-4_{Indol}). ^{13}C NMR (100 MHz, $CDCl_3$): δ 20.50, 20.57, 20.62 (4 CH_3CO), 48.4 (CH_2Ph), 61.2 (C-6_{Gal}), 67.00 (C-4_{Gal}, C-2_{Gal}), 71.60 (C-3_{Gal}), 75.30 (C-5_{Gal}), 83.90 (C-1_{Gal}), 107.90 (C-3_{Indol}), 110.90 (C-7_{Indol}), 121.20 (C-5_{Indol}), 122.14 (C-4_{Indol}), 122.20 (C-2_{Indol}), 125.20 (C-6_{Indol}), 126.40 (2C_{Ph}), 127.04 (C-$3a_{Indol}$), 127.30 (C_{Ph}), 128.60 (2C_{Ph}), 137.40 (C-$7a_{Indol}$), 139.50 (C_{Ph}), 160.20 (C-$5_{Oxad.}$), 161.20 (C-$2_{Oxad.}$), 169.60, 169.70, 170.10, 170.30 (4 CH_3*C*O). HRMS (EI): *m*/*z* calcd for $C_{31}H_{31}N_3O_{10}S$ [M^+] 637.1730, found 637.1752.

2-(2-Acetmido-3,4,6-tri-*O*-acetyl-2-deoxy-β-D-glucopyranosylsulfanyl)-5-(1-benzyl-1*H*-indol-2-yl)-1,3,4-oxadiazole (25)

Yield: (0.056 g, 9%) as a white powder. Mp 118–120 °C. TLC (2:3 *n*-hexane/ethyl acetate): R_f 0.41. ^{1}H NMR (400 MHz, acetone-d_6): δ 1.87, 1.97, 1.99 (3s, 12 H, 4 CO*C*H_3), 3.97-4.01 (m, 1 H, H-5_{GlcNAc}), 4.06 (dd, J_{gem} 12.4, $J_{5,6}$ < 1.0 Hz, 1 H, H-6_{GlcNAc}), 4.20 (dd, J_{gem} 12.4, $J_{5,6'}$ ≈ 5.2 Hz, 1 H, H-$6'_{GlcNAc}$), 4.26 (dd, $J_{1,2}$ 10.4, $J_{2,3}$ 9.6 Hz, 1 H, H-2_{GlcNAc}), 5.06 (dd, $J_{3,4}$ 9.6, $J_{4,5}$ 10 Hz, 1 H, H-4_{GlcNAc}), 5.38 (dd, $J_{2,3}$ ≈ $J_{3,4}$ 9.6 Hz, 1 H, H-3_{GlcNAc}), 5.76 (d, $J_{1,2}$ 10.4 Hz, 1 H, H-1_{GlcNAc}), 6.07 (s, 2 H, CH_2Ph), 7.11 (d, J 7.2 Hz, 2 H, Ph), 7.15-7.26 (m, 4 H, H-5_{Indol}, Ph), 7.26 (dd, $J_{5,6}$ 7.6, $J_{6,7}$ 8.4 Hz, 1 H, H-6_{Indol}), 7.41-7.45 (m, 2 H, H-3_{Indol}, NH_{GlcNAc}), 7.53 (d, $J_{6,7}$ = 8.4 Hz, 1 H, H-7_{Indol}), 7.74 (d, $J_{4,5}$ 8.0 Hz, 1 H, H-4_{Indol}). ^{13}C NMR (100 MHz, Acetone-d_6):

δ 20.40, 20.50, 20.60, 20.80 (4 CH_3CO), 48.80 (CH_2Ph), 53.80 (C-2$_{GlcNAc}$), 62.80 (C-6$_{GlcNAc}$), 69.40 (C-4$_{GlcNAc}$), 73.90 (C-3$_{GlcNAc}$), 77.00 (C-5$_{GlcNAc}$), 85.30 (C -1$_{GlcNAc}$), 108.30 (C-3$_{Indol}$), 111.90 (C-7$_{Indol}$), 121.90 (C-5$_{Indol}$), 122.80 (C-2$_{Indol}$, C-4$_{Indol}$), 125.70 (C-6$_{Indol}$), 127.30, 128.05, 128.10, 129.30 (C-3a$_{Indol}$, 5C$_{Ph}$), 138.95, 140.20 (C-7a$_{Indol}$, C$_{Ph}$), 161.5 (C-2$_{Oxad.}$ C-5$_{Oxad.}$), 169.90-170.50 (4 $COCH_3$). HRMS (EI): *m*/*z* calcd for $C_{31}H_{33}N_4O_9S$ [M^+] 637.1968, found 637.1940.

3-(2,3,4,6-Tetra-*O*-acetyl-β-D-glucopyranosyl)-5-(1-benzyl-1*H*-indol-2-yl)-1,3,4-oxadiazole-2(3*H*)-thione (26)

Yield: (0.226 g, 36%) as colorless crystals. Mp 203–205 °C. TLC (2:3 *n*-hexane/ethyl acetate): R_f 0.51. ^{1}H NMR (400 MHz, $CDCl_3$): δ 1.82, 2.02, 2.03, 2.05 (4s, 12 H, CH_3CO), 3.93 (m, 1 H, H-5$_{Glc}$), 4.12 (dd, J_{gem} 12.6, $J_{5,6}$ 2.2 Hz, 1 H, H-6$_{Glc}$), 4.23 (dd, J_{gem} 12.6, $J_{5,6'}$ 4.8 Hz, 1 H, H-6´$_{Glc}$), 5.16 (dd, $J_{3,4}$ 9.6, $J_{4,5}$ 10.0 Hz, 1 H, H-4$_{Glc}$), 5.36 (dd, $J_{2,3}$ 9.2, $J_{3,4}$ 9.6 Hz, 1 H, H-3$_{Glc}$), 5.55 (dd, $J_{1,2}$ 9.6, $J_{2,3}$ 9.2 Hz, 1 H, H-2$_{Glc}$), 5.76 (s, 2 H, CH_2Ph), 5.85 (d, $J_{1,2}$ 9.6 Hz, 1 H, H-1$_{Glc}$), 7.07 (m, 2 H, 2H$_{Ph}$), 7.16-7.27 (m, 4 H, H-5$_{Indol}$, 3H$_{Ph}$), 7.29-7.36 (m, 3 H, H-3$_{Indol,}$ H-6$_{Indol,}$ H-7$_{Indol}$), 7.69 (d, $J_{4,5}$ 8.0 Hz, 1 H, H-4$_{Indol}$). ^{13}C NMR (100 MHz, $CDCl_3$): δ 20.30, 20.50, 20.60 (4 CH_3CO), 48.40 (CH_2Ph), 61.60 (C-6$_{Glc}$), 67.60 (C-4$_{Glc}$), 68.90 (C-2$_{Glc}$), 73.30 (C-3$_{Glc}$), 74.65 (C-5$_{Glc}$), 83.00 (C -1$_{Glc}$), 109.90 (C-3$_{Indol}$), 110.80 (C-7$_{Indol}$), 120.60 (C-2$_{Indol}$), 121.40 (C-5$_{Indol}$), 122.50 (C-4$_{Indol}$), 125.90 (C-6$_{Indol}$), 126.60 (2C$_{Ph}$), 126.80 (C-3a$_{Indol}$), 127.50 (C$_{Ph}$), 128.70 (2C$_{Ph}$), 137.20 (C-7a$_{Indol}$), 139.90 (C$_{Ph}$), 154.40 (C-5$_{Oxad.}$), 168.80, 169.40, 169.90, 170.50 (4 CH_3CO), 176.90 (C=S). HRMS (EI): *m*/*z* calcd for $C_{31}H_{31}N_3O_{10}S$ [M^+] 637.1730, found 637.1729.

3-(2,3,4,6-Tetra-*O*-acetyl-β-D-galactopyranosyl)-5-(1-benzyl-1*H*-indol-2-yl)-1,3,4-oxadiazole-2(3*H*)-thione (27)

Yield: (0.175 g, 28%) as a yellowish white powder. Mp 98–100 °C. TLC (3:2 *n*-hexane/ethyl acetate): R_f 0.45. ^{1}H NMR (500 MHz, $CDCl_3$): δ 1.85, 1.99, 2.03, 2.08 (4s, 12 H, 4 CH_3CO), 4.07-4.10 (m, 1 H, H-6_{Gal}), 4.17-4.20 (m, 2 H, H-5_{Gal}, H-$6'_{Gal}$), 5.21 (dd, $J_{2,3}$ 10.5, $J_{3,4}$ 3.5 Hz, 1 H, H-3_{Gal}), 5.48 (dd, $J_{3,4} \approx J_{4,5}$ 3.5 Hz, 1 H, H-4_{Gal}), 5.79 (m, 3 H, H-2_{Gal}, CH_2Ph), 5.85 (d, $J_{1,2}$ 9.5 Hz, 1 H, H-1_{Gal}), 7.14-7.22 (m, 6 H, H-5_{Indol}, $5H_{Ph}$), 7.3 (dd, $J_{5,6}$ 7.4, $J_{6,7}$ 8.0 Hz, 1 H, H-6_{Indol}), 7.35 (s, 1 H, H-3_{Indol}), 7.39 (d, $J_{6,7}$ 8.0 Hz, 1 H, H-7_{Indol}), 7.70 (d, $J_{4,5}$ 8.0 Hz, 1 H, H-4_{Indol}). ^{13}C NMR (125 MHz, $CDCl_3$): δ 20.47, 20.51, 20.65 (4 CH_3CO), 48.40 (CH_2Ph), 61.10 (C-6_{Gal}), 66.50, 66.90 (C-2_{Gal}, C-4_{Gal}), 71.30 (C-3_{Gal}), 73.50 (C-5_{Gal}), 83.50 (C -1_{Gal}), 109.70 (C-3_{Indol}), 110.70 (C-7_{Indol}), 121.40 (C-5_{Indol}), 122.40 (C-2_{Indol}, C-4_{Indol}), 125.80 (C-6_{Indol}), 126.50 (C-$3a_{Indol}$), 126.90 ($2C_{Ph}$), 127.50 (C_{Ph}), 128.57 ($2C_{Ph}$), 137.20 (C-$7a_{Indol}$), 139.9 (C_{Ph}), 154.34 (C-$5_{Oxad.}$), 169.01, 169.81, 169.97, 170.35 (4 CH_3CO), 176.83 (C=S). HRMS (EI): *m*/*z* calcd for $C_{31}H_{31}N_3O_{10}S$ [M^+] 637.1730, found 637.1768.

3-(2-Acetamido-3,4,6-tri-*O*-acetyl-2-deoxy-β-D-glucopyranosyl)-5-(1-benzyl-1*H*-indol-2-yl)-1,3,4-oxadiazole-2(3*H*)-thione (28)

Yield: (0.439 g, 70%) as colorless needles. Mp 258–259 °C. TLC (2:3 *n*-hexane/ethyl acetate): R_f 0.58. ^{1}H NMR (400 MHz, DMSO-d_6): δ 1.63, 1.94,1.95, 2.00 (4s, 12 H, 4 $COCH_3$), 4.03 (m, 1 H, H-6_{GlcNAc}), 4.19 (m, 2H, H-5_{GlcNAc}, H-$6'_{GlcNAc}$), 4.25 (dd, $J_{1,2}$ 9.6, $J_{2,3}$ 10.0 Hz, 1H, H-2_{GlcNAc}), 4.95 (dd, $J_{3,4} \approx J_{4,5}$ 9.6 Hz, 1 H, H-4_{GlcNAc}), 5.40 (dd, $J_{2,3}$ 10.0, $J_{3,4}$ 9.6 Hz, 1 H, H-3_{GlcNAc}), 5.79, 5.87 (2d, J_{gem} 16 Hz, 2 H, CH_2Ph), 5.97 (d, $J_{1,2}$ 9.6 Hz, 1 H, H-1_{GlcNAc}), 7.17-7.28 (m, 6 H, H-5_{Indol}, $5H_{Ph}$), 7.36 (dd, $J_{5,6}$ 7.6, $J_{6,7}$ 8.4 Hz, 1 H, H-6_{Indol}), 7.40 (s, 1 H, H-3_{Indol}), 7.71 (d, $J_{6,7}$ 8.4 Hz, 1 H, H-7_{Indol}), 7.73 (d, $J_{4,5}$ 8.0 Hz, 1 H, H-4_{Indol}), 8.01 (d, J 9.2 Hz, 1 H, NH_{GlcNAc}). ^{13}C NMR (100 MHz, DMSO-d_6): δ 20.30, 20.40, 22.50 (4 $COCH_3$),

48.8 (CH_2Ph), 51.10 (C-2_{GlcNAc}), 61.40 (C-6_{GlcNAc}), 67.90, 72.10, 73.10 (C-3_{GlcNAc}, C-4_{GlcNAc}, C-5_{GlcNAc}), 83.00 (C -1_{GlcNAc}), 108.80 (C-3_{Indol}), 111.40 (C-7_{Indol}), 120.35 (C-2_{Indol}), 121.10 (C-5_{Indol}), 122.20 (C-4_{Indol}), 125.50 (C-6_{Indol}), 126.30 (C-$3a_{Indol}$), 126.90, 127.20, 128.80 (5C_{Ph}), 137.80 (C_{Ph}), 139.80 (C-$7a_{Indol}$), 153.30 (C-$5_{Oxad.}$), 169.30, 169.50, 169.60, 169.70 (4 $COCH_3$), 175.8 (C=S). HRMS (EI): *m*/*z* calcd for $C_{31}H_{33}N_4O_9S$ [M^+] 637.1968, found 637.1850.

General procedure for thermal conversion of 23–25 into **26–28**

The appropriate *S*-glycoside **23–25** (0.13 g, 0.2 mmol) was heated as described for the thermal conversion of **5–7** into **8–10** and the product was purified by flash chromatography (*n*-hexane/ethyl acetate, 2:3) to afford the corresponding *N*-glycoside **26**–**28** in yields: (0.092 g, 71%), (0.085 g, 65%) and (0.101 g, 78%).

General procedure for synthesis of compounds 29, 30

The appropriate glycoside **26–27** (0.13 g, 0.2 mmol) was treated with ammonia as described for the synthesis of **11–13**, and the residue was purified by recrystallization from EtOH.

1-(1-Benzyl-1*H*-indol-2-ylcarbonyl)-2-(β-D-glucopyranosyl)thiosemicarbazide (29)

Yield: (0.081 g, 82%) as colorless needles. Mp 242–243 °C. TLC (9:1 CH_2Cl_2/MeOH): R_f 0.26. ^1H NMR (400 MHz, DMSO-d_6 + D_2O): δ 3.08-3.54 (m, 5 H, H-2_{Glc}, H-3_{Glc}, H-4_{Glc}, H-5_{Glc}, H-6_{Glc}), 3.64 (dd, J_{gem} 10.4, $J_{5,6'}$ < 1.0 Hz, 1H, H-$6'_{Glc}$), 5.68 (d, J_{gem} 15.6 Hz, 1 H, C*H*HPh), 5.81 (d, J_{gem} 15.6 Hz, 1 H, CH*H*Ph), 6.32 (br. d, 1 H, H-1_{Glc}), 7.16-7.29 (m, 7 H, H-5_{Indol}, H-6_{Indol}, 5H_{Ph}), 7.49-7.70 (m, 2 H, H-3_{Indol}, H-7_{Indol}), 7.70 (d, $J_{4,5}$ 7.8 Hz, 1H, H-4_{Indol}). ^{13}C NMR (100 MHz, DMSO-d_6 + D_2O): δ 47.40 (CH_2Ph), 60.70 (C-6_{Glc}), 69.70, 70.50, 75.80, 78.70 (C-2_{Glc}, C-3_{Glc}, C-4_{Glc}, C-5_{Glc}), 86.80 (C-1_{Glc}), 108.50 (C-3_{Indol}), 111.30 (C-7_{Indol}), 120.70 (C-5_{Indol}), 122.10 (C-4_{Indol}), 124.60 (C-6_{Indol}), 125.67, 127.00, 127.10 (C-2_{Indol}, C-

3a$_{Indol}$, 3C$_{Ph}$), 128.40 (2C$_{Ph}$), 138.40 (C-7a$_{Indol}$), 138.5 (C$_{Ph}$), 163.50 (C=O), 184.60 (C=S). HRMS (ESI): *m/z* calcd for $C_{23}H_{27}N_4O_6S$ [M+H$^+$] 487.1651, found 487.1630.

5-(1-Benzyl-1*H*-indol-2-yl)-2-(β-D-glucopyranosyl)-2*H*-1,2,4-triazoline-3-thione monohydrate (30)

Yield: (0.077 g, 78%) as a colorless needles. Mp 251–253 °C. TLC (9:1 CH_2Cl_2/MeOH): R_f 0.23, ^{1}H NMR (300 MHz, DMSO-d_6): δ 3.48-3.61 (m, 4 H, H-3$_{Gal}$, , H-5$_{Gal}$, H-6$_{Gal}$, H-6´$_{Gal}$), 3.78 (dd, $J_{3,4}$ = $J_{4,5}$ 3.6 Hz, 1 H, H-4$_{Gal}$), 4.18-4.24 (m, 1 H, H-2$_{Gal}$), 4.59 (d, *J* 3.9 Hz, 1 H, OH$_{Gal}$ *D_2O exchangeable*), 4.65 (dd, *J* 5.1, *J* 5.7 Hz, 1 H, OH$_{Gal}$ *D_2O exchangeable*), 4.95 (d, *J* 5.7 Hz, 1 H, OH$_{Gal}$ *D_2O exchangeable*), 5.01 (d, *J* 5.4 Hz, 1H, OH$_{Gal}$ *D_2O exchangeable*), 5.56 (d, 1 H, $J_{1,2}$ 8.7 Hz, H-1$_{Gal}$), 5.88 (s, 2 H, CH_2Ph), 7.10-7.33 (m, 8 H, H-3$_{Indol}$, H-5$_{Indol}$, H-6$_{Indol}$, 5H$_{Ph}$), 7.67 (d, $J_{4,5}$ = $J_{6,7}$ 8.4 Hz, 2 H, H-4$_{Indol}$, H-7$_{Indol}$), 14.12 (s, 1 H, NH$_{Triazol}$ *D_2O exchangeable*). ^{13}C NMR (100 MHz, DMSO-d_6 + D_2O): δ 48.18 (CH_2Ph), 61.10 (C-6$_{Gal}$), 68.67, 68.75 (C-2$_{Gal}$, C-4$_{Gal}$), 74.29, 78.50 (C-3$_{Gal}$, C-5$_{Gal}$), 84.93 (C-1$_{Gal}$), 106.83 (C-3$_{Indol}$), 111.80 (C-7$_{Indol}$), 121.49 (C-5$_{Indol}$), 122.29 (C-4$_{Indol}$), 124.14 (C-2$_{Indol}$, C-6$_{indol}$), 127.24, 128.04. 129.16 (C-3a$_{Indol}$, 5 C$_{Ph}$), 138.52, 139.34 (C-7a$_{Indol}$, C$_{Ph}$), 144.18 (C-5$_{Triazol}$), 168.48 (C=S). HRMS (FAB): *m/z* calcd for $C_{23}H_{25}N_4O_5S$ [M − H_2O + H$^+$] 469.1545, found 469.1563.

References

1. El Ashry, E. S. H.; El Tamany, E. H.; Aly, M. R. E.; Boraei, A. T. A. *Synlett.* **2010**, *15*, 2247–2250.

2. Hirmath, S. P.; Hirmath, D. M.; Purohit, M. G. *Indian J. Chem.* **1983**, *22B*, 571–576.

3. Robba, M.; Maume, D.; Lancelot, J. C., *Bull. Soc. Chim. Fr.* **1977**, *3-4*, 333–336.
